# DBA‐DeepLab: Dual‐Backbone Attention‐Enhanced DeepLab V3+ Model for Plant Disease Segmentation

**DOI:** 10.1002/fsn3.70668

**Published:** 2025-07-21

**Authors:** Neha Sharma, Sheifali Gupta, Fuad Ali Mohammed Al‐Yarimi, Yazeed Yasin Ghadi, Salil Bharany, Ateeq Ur Rehman, Seada Hussen

**Affiliations:** ^1^ Chitkara Institute of Engineering and Technology Chitkara University Rajpura Punjab India; ^2^ Applied College of Mahail Aseer King Khalid University Saudi Arabia; ^3^ Department of Computer Science and Software Engineering Al Ain University Abu Dhabi UAE; ^4^ School of Computing Gachon University Seongnam‐si Republic of Korea; ^5^ Department of Electrical Power Adama Science and Technology University Adama Ethiopia

**Keywords:** CBAM module, DBA‐DeepLab, DeepLab V3+, dual backbone, EfficientNet B3, plant leaves, ResNet 50, segmentation

## Abstract

Accurate and efficient plant disease segmentation is crucial for early diagnosis and precision agriculture. In this study, we propose a DBA‐DeepLab model, i.e., a Dual‐Backbone Attention‐Enhanced DeepLab model, which integrates DeepLabV3+ with dual backbones of ResNet‐50 and EfficientNet‐B3 and a Convolutional Block Attention Module (CBAM) for improved plant disease segmentation. The integration of multi‐scale feature extraction, attention mechanisms, and edge preservation with the Sobel filter enhances the ability of the model to focus on disease‐affected regions with more accuracy and reduce false positives and false negatives. The model was trained and validated using the PlantDoc dataset with a batch size of 32, Adam optimizer, and 50 epochs for better convergence and generalization. Experimental results show that the proposed DBA‐DeepLab outperforms DeepLabV3+ with EfficientNet‐B3 encoder, DeepLabV3+ with ResNet‐50 encoder, and DeepLabV3+ with dual encoder (EfficientNet‐B3 and ResNet‐50) in terms of segmentation parameters. The proposed model yields 99.35% accuracy, a 91.48% Dice coefficient, an 85.85% IoU coefficient, 96.78% precision, and 100% recall, outperforming the state‐of‐the‐art. Grad‐CAM visualization was applied to validate the model's interpretability, affirming its capacity to highlight disease‐affected regions and avoid background noise. Comparative analyses with these DeepLabV3+ variants support the improved generalization, segmentation accuracy, and robustness of the proposed model. These results show that DBA‐DeepLab is an extremely efficient and scalable solution for plant disease segmentation, with potential applications in smart farming, automatic disease detection, and precision agriculture.

## Graphical abstract

This study introduces DBA‐DeepLab, a novel plant disease segmentation model that integrates dual backbones—ResNet‐50 and EfficientNet‐B3—with a Convolutional Block Attention Module (CBAM) and Sobel filtering for enhanced accuracy. Trained on the PlantDoc dataset, the model achieves superior performance metrics, including 99.35% accuracy and 100% recall, making it highly effective for precision agriculture and automated disease detection.

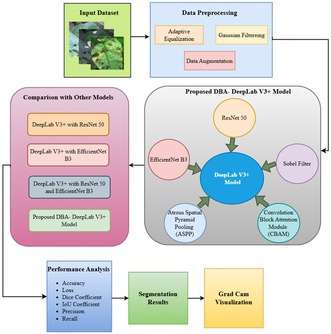

## 1 Introduction

Agriculture is at the heart of global food security and economic stability, and plant health is a major component of agricultural productivity (Masood et al. 2016). Plant diseases significantly impact crop yields, causing enormous financial losses and contributing to food shortages worldwide (Singh and Misra 2017). Detection and accurate identification of plant diseases at an early stage are crucial for timely intervention and effective disease management. The conventional detection approaches of plant diseases are predominantly manual inspection by experts, which is time‐consuming, prone to human mistakes, and feasible for massive agricultural cultivation. In recent times, the strides in artificial intelligence and deep learning have opened a new avenue towards automated plant disease detection and segmentation, providing solutions that are efficient, scalable, and precise to modern farming needs (Barbedo 2017; Ferentinos 2018). Root pathogens cause plant disease through the infections of fungi, bacteria, viruses, and even nematodes that affect plants' different components, like leaves, stems, and fruits. Some diseases affect specific parts of plants and can potentially impact the crop yields. In some cases, they compromise the quality and value of these crops, potentially resulting in considerable losses for the farmer economically and food deficiency for the whole society. For example, late blight of potatoes and tomatoes can destroy entire fields if not recognized immediately, thereby needing management (Gurrala et al. 2019; Wang et al. 2019). Similarly, rusts in wheat have had a historically consistent heavy use for loss of yield in cereal crops. Plant diseases also have an important economic impact. The Food and Agriculture Organization stated that around 10%–16% of the total global harvest is lost annually, accounting for the approximate loss amounting to $220 billion every year by the action of plant pests and diseases. Every year, crop loss resulting from pathogens that originally belonged outside US territory accounted for around $21 billion of the US economy (Bell and Dee 2019). These data further amplify the urgent requirement to develop successful disease management approaches that protect food security and agricultural livelihoods. Traditionally, plant diseases have been detected based on visual examination. This approach relies on qualified experts evaluating symptoms like leaf spots, discoloration, wilting, or even mold appearance. However, this method has several drawbacks (Gurrala et al. 2019; Wang et al. 2019; Bell and Dee 2019; Kaushik et al. 2020). Visual assessments are labor‐intensive and time‐consuming; hence, their use is impractical at a large‐farm scale. Some specific pathogens are diagnosed through microscopy and mycological analyses (Verma et al. 2020). These methods, however, require specialized equipment and expertise, further limiting their applicability in field conditions. With growing concerns over the limitation of the traditional approach in the detection of plant diseases, researchers have gained more interest in the development of automated methods. Promising advancement has been noted with artificial intelligence and deep learning in this field (Kaushik et al. 2020; Verma et al. 2020; Wspanialy and Moussa 2020). Machine‐learning algorithms can be trained to identify the symptoms of the disease through images, and identification can be fast and accurate. The convolutional neural networks (CNNs) have also performed very well in disease classification of plants based on leaf images. The models can spot intricate patterns and image features that might go undetected to the naked eye, thereby refining the accuracy of disease identification (Karthik et al. 2020; Ahmad et al. 2020). Recent advancements in deep learning have significantly enhanced image segmentation tasks across various domains (Wspanialy and Moussa 2020; Karthik et al. 2020; Ahmad et al. 2020). Among the notable architectures, U‐Net has emerged as a prominent model for image segmentation tasks (Feng et al. 2025). Its encoder‐decoder structure with skip connections allows for the effective capture of contextual information and precise localization, making it particularly suitable for applications with limited training data. Furthermore, the integration of attention mechanisms into deep‐learning models has enhanced their ability to focus on relevant regions of an image, suppressing irrelevant information and improving segmentation performance, especially in complex or noisy environments (Liu et al. 2024).

The proposed work introduces a new DeepLabV3 + ‐based segmentation model with a dual‐backbone architecture using ResNet‐50 and EfficientNet‐B3, an attention mechanism, and multi‐scale feature extraction techniques. Utilization of the dual‐backbone network, leveraging the residual learning ability of ResNet‐50 and depth‐width scaling optimization of EfficientNet‐B3, improves feature extraction. The use of the Convolutional Block Attention Module (CBAM) is utilized for spatial and channel attention for better feature selection and disease localization. Utilization of Atrous Spatial Pyramid Pooling (ASPP) enhances multi‐scale feature learning to detect disease lesions with different sizes and intensities. An extensive performance comparison of the introduced model with other existing segmentation models is based on various quantitative measures like intersection over union (IoU), Dice coefficient, precision, recall, and accuracy. Aside from theoretical contributions, the implication of this work provides practical application for precision agriculture and smart farming applications. By enhancing the precision and stability of plant disease segmentation, the model can enable real‐time disease detection in fields, allowing farmers to respond in a timely manner and minimize crop losses. The incorporation of AI‐based disease monitoring systems into mobile apps, drone monitoring, and Internet of Things (IoT) platforms can transform plant health evaluation, offering automated, low‐cost, and scalable solutions for sustainable agriculture. In addition, the implementation of deep learning‐based segmentation models in agricultural science can contribute to the creation of new disease‐resistant crop varieties and enhance plant pathology research through high‐resolution, annotated data sets for disease detection.

The major contributions of this research work are as follows:

- The integration of ResNet‐50 and EfficientNet‐B3 as dual backbones in the DBA‐DeepLab model enhances feature extraction by leveraging residual learning and optimized depth‐width scaling, ensuring superior representation of disease‐affected regions and improving segmentation performance.
- The incorporation of the CBAM refines feature selection by applying spatial and channel attention, enabling the model to focus on disease‐specific areas while suppressing irrelevant background information, thus improving disease localization and segmentation precision.
- The use of ASPP captures contextual information at multiple scales, allowing for the accurate detection of lesions of varying sizes and intensities, making the model robust to different plant disease patterns.
- The proposed DBA‐DeepLab model is rigorously evaluated against DeepLabV3+ with EfficientNet‐B3, DeepLabV3+ with ResNet‐50, and DeepLabV3+ with both EfficientNet‐B3 and ResNet‐50 using multiple quantitative metrics such as IoU, Dice coefficient, precision, recall, and accuracy, demonstrating its superior segmentation capabilities.

The rest of this paper is structured as follows: Section 2 shows the related work of the current deep learning‐based segmentation algorithms. Section 3 outlines the proposed methodology, including the dataset, the DBA‐DeepLab model that is proposed in this paper, its dual‐backbone architecture, the CBAM attention mechanism, and ASPP‐based multi‐scale feature extraction. Section 4 addresses results and discussion, which shows the experimental setup, performance evaluation, and comparative analysis of the proposed model and other models. Section 6 shows the conclusions and future work of the paper.

## 2 Related Work

Plant disease detection and classification are essential for maintaining agricultural productivity and food security. Several image processing and deep learning‐based methods have been suggested to improve the accuracy and efficiency of plant disease detection. This literature review discusses various segmentation and classification methods employed in plant disease detection, their strengths, weaknesses, and future scope for improvement. Kaushik et al. (2020) proposed a model for the detection of tomato leaf disease using ResNet‐50 with the application of transfer learning. In their work, data augmentation methods were used to enhance the model's ability to generalize in the field. Augmenting the dataset size, they achieved a classification accuracy of 97%, demonstrating the significance of transfer learning in the detection of plant diseases. Verma et al. (2020) investigated AlexNet, SqueezeNet, and Inception V3 architectures for the grading of tomato late blight disease. They considered three stages for the segmentation of images and tried both transfer learning and feature extraction approaches. For the two separate conditions, their results reported a maximum accuracy obtained by AlexNet at 89.69% and 93.4%, respectively. Wspanialy and Moussa (2020) introduced an advanced computer vision‐based system that allows them to detect and classify tomato diseases successfully. Their model was learned using altered PlantVillage datasets, thereby making the system able to detect multiple plant diseases. The paper further demonstrated that automated detection of plant diseases was feasible. However, most significantly, it highlighted the need to integrate per‐leaf severity estimation in the process so as to improve proper disease management and crop monitoring. Karthik et al. (2020) also suggested two deep‐learning models for the detection of tomato leaf disease. Their first architecture depends on residual learning to enhance feature extraction, and their second model utilizes an attention mechanism for enhancing classification accuracy. Their models demonstrated 98% accuracy in 5‐fold cross‐validation testing, which reflects the possibility of deep learning application in plant disease detection. Ahmad et al. (2020) evaluated several CNN architectures, such as VGG‐16, VGG‐19, ResNet, and Inception V3, for the classification of tomato diseases. Their work used field‐gathered and lab‐simulated datasets and found deep‐learning models to perform superior under laboratory conditions. Gadekallu et al. (2021) investigated machine‐learning methods for image classification of tomato diseases. The authors used a hybrid method, which is the use of PCA and the Whale Optimization Algorithm for feature extraction. The proposed approach reduced dimensionality without losing important disease‐specific features, enhancing computational efficiency. Dayang and Meli (2021) contrasted various segmentation techniques, such as k‐means clustering, canny edge detection, and k‐nearest neighbor (KNN) for plant disease classification. They proved through their study that the k‐means cluster performed better than other approaches in segmentation. Atila et al. (2021) used EfficientNet architectures for plant disease classification and compared them with other deep‐learning models. Their models, specifically EfficientNet‐B5 and EfficientNet‐B4, achieved extremely high classification accuracies of 99.91% and 99.97%, respectively. Their work focused on the use of deep architectures of the convolutional network in achieving improvement in plant disease detection; with that, it showed how EfficientNet outperformed others by extracting and classifying better. The obtained results proved that lightweight and super‐efficient architectures like EfficientNet can be used in mobile and edge computing for real‐time disease identification purposes.

Chen et al. (2021) proposed an enhanced artificial neural network (ANN) along with a new approach toward segmentation and classification. Their hybrid model showed an average accuracy of 93.75% in the detection of plant diseases with complex backgrounds. In a few cases, the recall was 100%, meaning that almost all of the samples from plant diseases had been correctly captured. Shoaib et al. (2022) proposed a deep learning‐based system for tomato plant disease detection from leaf images. They employed a modified U‐Net model for disease‐affected area segmentation and InceptionNet for classification. The work attained high accuracy in segmentation and classification, showing the efficiency of using these models together for plant disease detection. Khan et al. (2022) suggested an end‐to‐end semantic leaf segmentation model for the classification of plant diseases. Their approach has advanced the application of a deep convolutional neural network, which performs better in segmenting and locating regions of diseased areas on the leaves of the plant. It is designed to analyze images of plant leaves, thus separating healthy and diseased regions through the application of methods in semantic segmentation. Zhang and Zhang (2023) introduced a better U‐Net model that they termed MU‐Net for plant‐diseased leaf image segmentation. This method incorporates residual blocks (Resblock) and residual paths (Respath) to address issues like gradient vanishing and enhance feature extraction capability. MU‐Net‐based models performed better than traditional U‐Net models in segmenting diseased leaf images, leading to effective diagnosis of the diseases. Such an enhancement in the segmentation enables better health monitoring and maintenance of plants. Mzoughi and Yahiaoui (2023) introduced a new technique for classifying plant disease where the task of disease identification is separated from leaf species identification. In this method, the system can identify diseases in new plant species outside the training set. Chillakuru et al. (2024) presented a CNN model‐based PLDC with an optimized nine‐layered architecture. Adaptive Fuzzy C‐Means (A‐FCM) were utilized in image segmentation by their approach to detect abnormal regions on plant leaves with high accuracy. The research also involved the utilization of a hybrid heuristic optimization algorithm, Hybrid Leader Cat Swarm Optimization (HLCSO), to optimize segmentation and classification parameters. Krishnan et al. (2022) proposed a cost‐effective, robust image processing‐based approach for banana plant disease detection. They applied hybrid fuzzy C‐means clustering‐based segmentation with subsequent color, shape, and property‐based feature extraction. The system identified the diseases black Sigatoka, yellow Sigatoka, banana bacterial wilt, and wilted leaves with deep learning‐based classification models. Singh et al. (2022) explored the use of AlexNet for maize plant leaf disease detection using the PlantVillage dataset for training and testing. They have worked on two significant maize‐based leaf diseases—leaf spot‐derived diseases and common rust‐derived diseases. Their findings highlighted the potential of CNN‐based feature extraction for real‐time disease detection and, once again, illustrated the potential of deep‐learning models to be used as part of effective tools for assisting farmers in detecting diseases as well as protecting crops. Elaraby et al. (2022) suggested a deep learning‐based system for the detection of plant diseases in various types of crops such as wheat, cotton, grape, corn, and cucumbers. The study employed a blend of AlexNet and particle swarm optimization (PSO) to train and tune the model. The study proved the applicability of deep learning in large‐scale, multi‐crop disease detection. It highlighted the requirement for strong optimization techniques to improve model generalization in real‐world applications. Badiger and Mathew (2023) proposed a deep learning‐based approach for tomato disease detection and classification using a batch‐normalized eLu‐enhanced AlexNet (DbneAlexNet). The proposed segmentation technique used U‐Net, which was trained using a Gradient‐Golden Search Optimization (G‐GSO) algorithm, significantly improving segmentation precision. Wang, Ding, et al. (2023) proposed a new pear leaf disease segmentation model called MFBP‐UNet, designed on the architecture of U‐Net. The model integrated the MFE module with a BATok‐MLP module, incorporating dynamic sparse attention mechanisms into feature representation. Moreover, the paper added diffusion models for data augmentation purposes to improve the robustness of training. Experiment results showed that remarkable improvements in segmentation performance occurred since the proposed approach demonstrated an MIoU score of 86.15% and a Dice coefficient of 0.922, along with high precision and recall rates. Kaur et al. (2024) proposed a hybrid Deep Segmentation Convolutional Neural Network for object detection in plant disease segmentation. It was developed by combining pretrained models of U‐Net and SegNet, which helped in the instance segmentation of diseased areas in the tomato plant. This hybrid segmentation approach shows superior performance as compared with modified U‐Net, M‐SegNet, and modified U‐SegNet models at a processing accuracy of 98.24%, handling 1004 images within a processing time of 30 ns. Polly and Devi (2024) proposed a multi‐stage plant disease detection framework utilizing YOLOv8 for region of interest identification, DeepLabV3+ for background removal, and CNN for disease classification. Their model achieved a training accuracy of 96.97% and a validation accuracy of 92.89%, with more segmentation using U‐Net achieving an impressive accuracy of 99%. The study also proposed a novel severity assessment stage that produced action‐oriented recommendations for disease management. Dai et al. (2024) proposed a new semantic segmentation algorithm, AISOA‐SSformer, inspired by Transformer architectures for the purpose of detecting pests and diseases in rice leaves. This revolutionary effect indicates how the union of attention‐grabbing transformers and deep learning facilitates more precise and adaptable detection of plant diseases.

## 3 Proposed Methodology

The proposed DBA‐DeepLab model is based on DeepLabV3+ (Wang, Zhang, et al. 2023) with dual‐backbone architecture, CBAM attention, and a Sobel filter to learn improved features and enhance the segmentation accuracy. The dual‐backbone structure is a combination of EfficientNet‐B3 and ResNet‐50 with complementary strengths. EfficientNet‐B3 offers lightweight but strong depth‐width scaling for effective feature extraction, and ResNet‐50 enhances residual learning with the extraction of fine patterns in disease regions. The fusion assists the model in learning multi‐scale contextual features necessary for the effective segmentation of diseased‐infected parts of the leaves. For better feature selection, the CBAM is brought into the network. CBAM employs spatial and channel attention mechanisms, so the model can pay attention to the most significant areas of the plant leaf and not the less significant background information. Attention fine‐tuning based on attention improves the disease lesion localization, leading to more accurate segmentation results.

The model further employs ASPP to promote multi‐scale feature learning by obtaining context information at a range of different resolutions, aiding in the identification of disease areas of different intensity and size. The Sobel filter is the other significant feature added to the proposed model and is employed as an edge detector to enhance the detection of boundaries. This filter emphasizes the edge of the infected areas so that the model can distinguish more clearly between infected and healthy areas. By incorporating the Sobel filter, the network is better able to detect fine‐grained disease patterns, reducing misclassification and improving segmentation accuracy. The combined effect of the double backbone structure, CBAM attention block, ASPP multi‐scale feature extractor, and Sobel edge detection all contribute to a very robust segmentation model.

**FIGURE 1:**
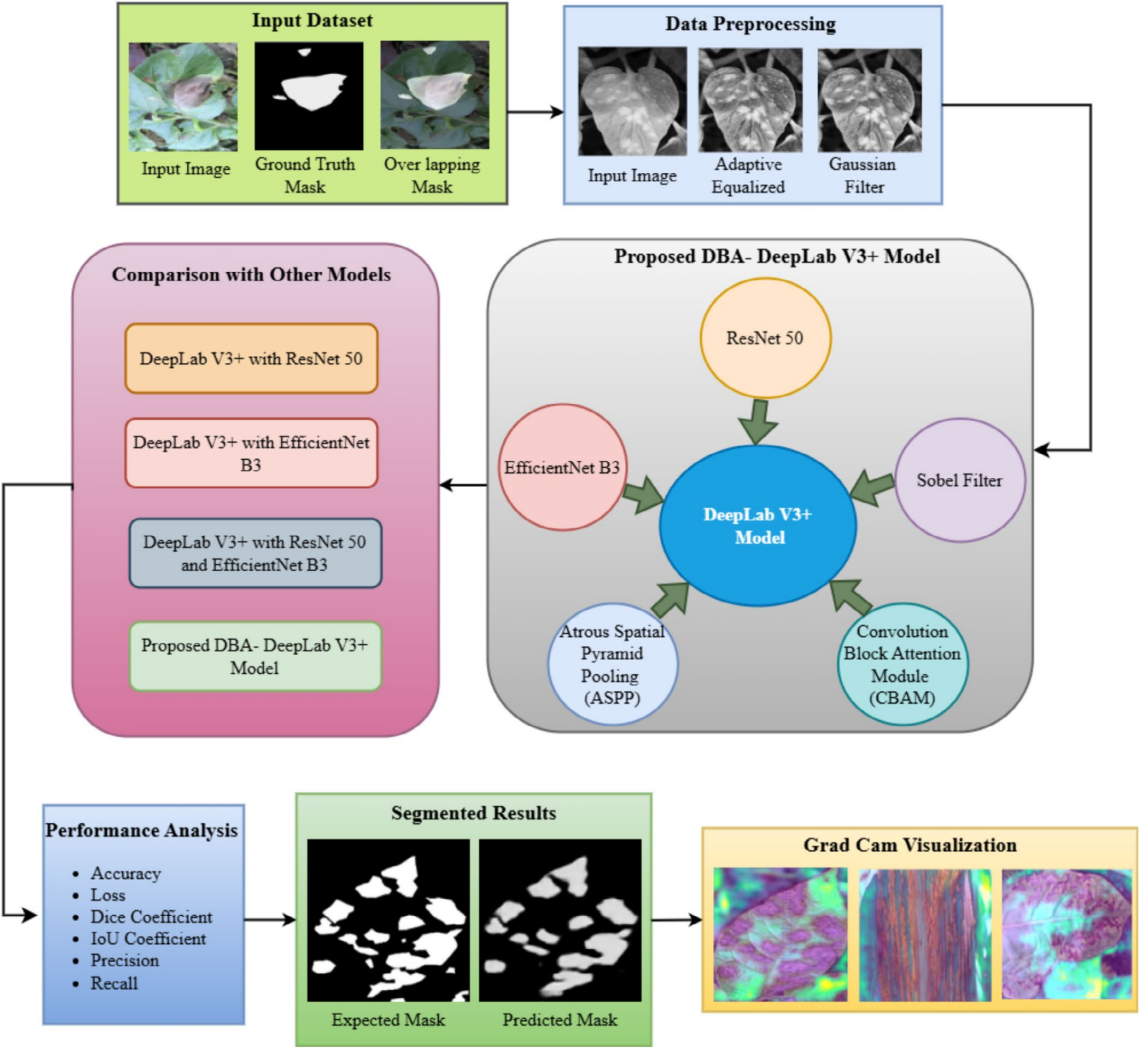
Proposed methodology for plant leaf disease segmentation.

Figure 1 describes the proposed Dual‐Backbone Attention‐Enhanced DeepLab (DBA‐DeepLab) architecture for plant disease segmentation, stating its different components, benchmark architectures, and performance evaluation. Segmentation is performed starting with the input data, in which images of plant leaves showing signs of diseases are gathered. The data consists of input images, ground‐truth masks, and overlapping masks such that the model is trained through supervised learning to segment diseased areas properly. For improved segmentation accuracy, the images are preprocessed through a data preprocessing process, where adaptive histogram equalization is applied to enhance contrast and Gaussian filtering to blur the images and remove noise such that effective feature extraction is guaranteed. The segmentation model structure emphasizes the essential building blocks of the DBA‐DeepLab model in question, which is blessed with dual backbones—ResNet‐50 and EfficientNet‐B3—to carry out higher‐level feature extraction. The deep residual learning architecture of ResNet‐50 facilitates hierarchical feature extraction, whereas EfficientNet‐B3's scaling policy for optimization enhances computational efficiency and representation learning. Both these backbones are merged to leverage both deep contextual understanding and light feature extraction so that the model is incredibly efficient in segmenting plant disease symptoms highly accurately.

Along with the two‐backbone architectures, CBAM is also introduced in the model to further optimize feature selection utilizing spatial and channel attention mechanisms. This enhances the model's focus on disease‐infected regions and lessens the impact of unwanted background data. Also, the ASPP module is employed to extract multi‐scale contextual information so that the model can segment different sizes and intensities of disease symptoms. The Sobel filter is incorporated to preserve the edge details and avoid segmentation error by ambiguous boundaries, while the model should properly distinguish between healthy and diseased areas. The model is rigorously compared with three implementations of DeepLabV3+, including DeepLabV3+ with ResNet‐50, DeepLabV3+ with EfficientNet‐B3, and DeepLabV3+ with both backbones, for a fair performance evaluation. The comparison part of Figure 1 graphically distinguishes these models and their structural patterns, showing how the proposed DBA‐DeepLab model performs better than others. The performance analysis part compares the quality of segmentation based on various quantitative parameters like accuracy, loss, Dice coefficient, IoU coefficient, precision, and recall for a complete analysis of the reliability and robustness of the model. The divided results section depicts the visual comparison between the expected mask and the predicted mask (ground truth). It exhibits the ability of the model to demarcate correctly disease‐affected regions. The high rate of similarity between the expected and actual masks shows the capability of the model for precise segmentation, which is crucial in precision agriculture and autonomous plant disease monitoring. Gradient‐weighted class activation mapping (Grad‐CAM) visualization is subsequently used to obtain the model's decision‐making process. Grad‐CAM visualization focuses on highlighting the regions the model is focusing on during prediction so that it can efficiently identify symptoms of the disease and ignore background noise. The Grad‐CAM results confirm that the DBA‐DeepLab model is capable of localizing disease patterns efficiently, thus proving its ability to provide accurate and interpretable segmentation outcomes.

### 3.1 Input Dataset

The Plant Leaf Disease Segmentation Dataset is a specialized dataset for semantic segmentation of plant leaf diseases. It can be utilized to train deep‐learning models for researchers and practitioners in order to automate disease detection. It consists of 588 plant‐diseased leaf images and 588 segmentation masks, which are precise annotations employed to label the diseased area from the background. The images are from the PlantDoc dataset, a widely used dataset for plant disease segmentation and classification tasks, which is proposed to assist deep learning‐based approaches toward automated disease diagnosis. The dataset includes a diverse set of images of plants' leaves showing a wide variety of crop species and respective diseases. These photographs are taken in real environments with the dynamics of lighting, background, and photo quality, and hence are perfectly suited for training accurate machine‐learning models. The dataset of PlantDoc contains images of various crops infected with many diseases like apple scab, apple rust, bell pepper leaf spot, corn leaf blight, potato early blight, etc.

**FIGURE 2:**
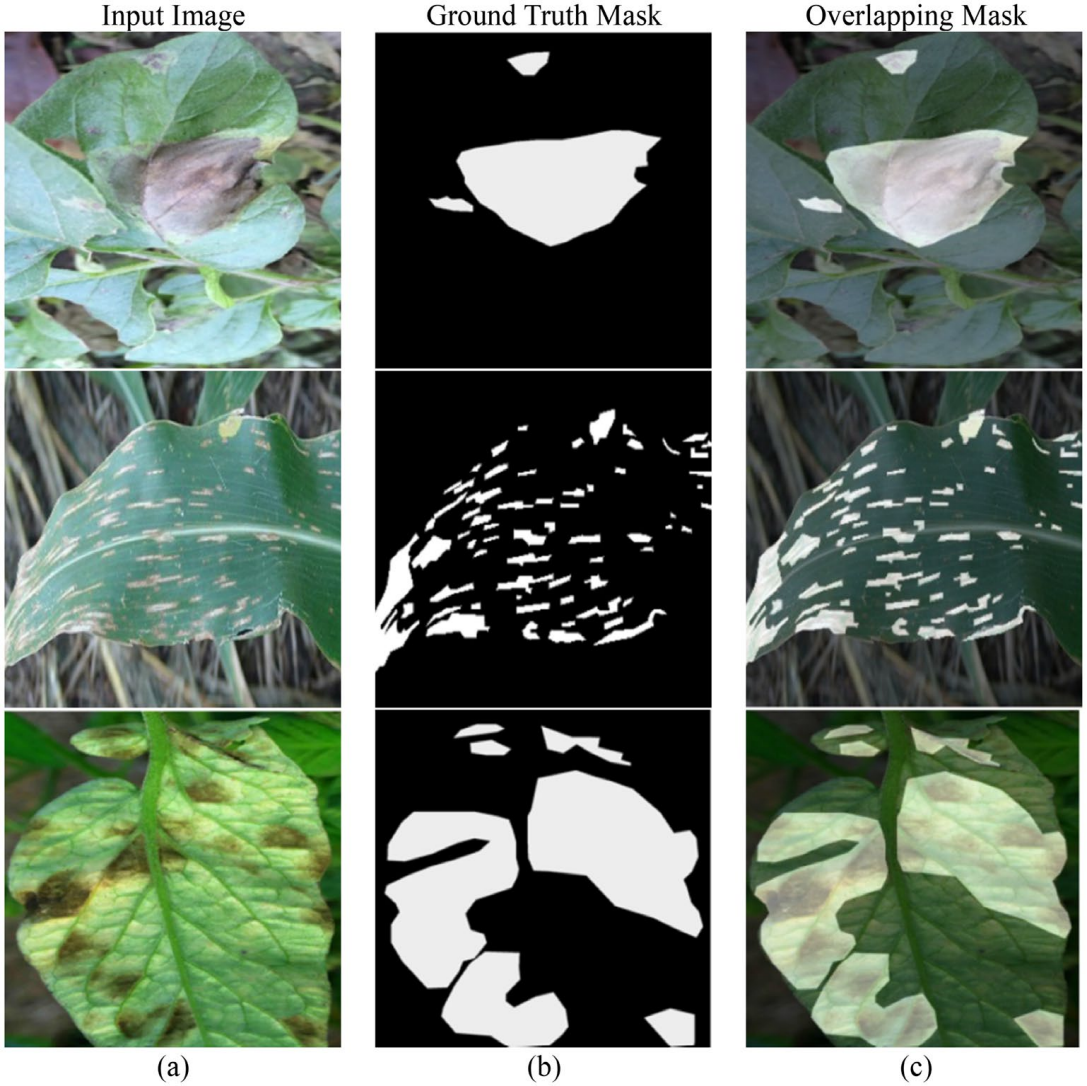
Sample images from the PlantDoc dataset with their corresponding masks: (a) Input image, (b) Ground truth mask, and (c) Overlapping mask.

Figure 2 illustrates the diseased plant leaf segmentation process with a combination of input images, the ground‐truth masks, and overlapping masks. It has three columns: the input image (left column), ground‐truth segmentation mask (center column), and the overlapping mask (right column). Each row is for an individual diseased plant leaf, and it shows the segmentation process for different samples. The left column (input image) contains real images of plant leaves infected with various diseases. The images possess multiple types of infections, i.e., apparent color changes, lesions, and necrotic spots. The change in background, lighting, and orientation of the leaves reflects the challenge of segmenting the infected areas exactly under actual farm conditions. The right column (ground‐truth mask) contains binary segmentation masks of the input images. These hand‐annotated masks mark the disease‐infected regions in white, with black areas indicating the background and healthy parts of the leaf. These masks can be employed as ground‐truth annotations for training and testing deep‐learning models, enabling supervised learning to obtain precise disease segmentation. The third column (overlapping mask) shows an overlaid visualization where the ground‐truth mask is overlaid on the original input image. This overlay provides a qualitative impression of how closely the segmentation mask aligns with the actual diseased regions. It helps to assess the quality of the annotation process visually and serves as a reference for judging the performance of machine‐learning models.

### 3.2 Dataset Preprocessing

Preprocessing of the dataset is needed to improve the quality of the input images and the performance of the segmentation model. The raw plant leaf images have variations in lighting conditions, background noise, image resolution, and disease intensity, which can negatively impact the accuracy of deep‐learning models. To counter such issues, a series of preprocessing steps is applied to normalize and prepare the dataset for effective disease segmentation. Image resizing and normalization are done as the first steps to gain homogeneous input sizes and pixel intensity levels such that the dataset will be compatible with deep learning. Computational complexity is reduced without loss of relevant disease‐specific information using this method. Adaptive histogram equalization is utilized to enhance contrast and improve diseased area representation. The technique reallocates pixel intensities in the image in such a way as to allow the model to identify diseased areas more effectively, separated from healthy foliage material. The proposed DBA‐DeepLab model is specifically designed to handle challenging plant leaf images with complex natural backgrounds and lighting variations, as shown in Figure 2a. These real‐field images introduce substantial background noise that can interfere with accurate disease segmentation. To address this, the model incorporates the following multi‐level robustness strategy:

The Sobel filter is employed after Gaussian filtering, which smooths the image and suppresses high‐frequency noise. This two‐step preprocessing reduces the sensitivity of edge detection to background clutter, helping the model delineate infected regions more clearly.

Additionally, the CBAM attention module is integrated into the model architecture. CBAM applies channel and spatial attention, enabling the network to prioritize relevant disease‐affected features and suppress irrelevant background noise. As described in both the Proposed Methodology and Dataset Preprocessing sections, this mechanism enhances the model's focus on diseased regions, especially under visually complex conditions.

As seen in Figure 2a, the input images reflect the complexity typical of real‐world agricultural settings, and the proposed architecture demonstrates strong segmentation performance despite such visual interference.

**FIGURE 3:**
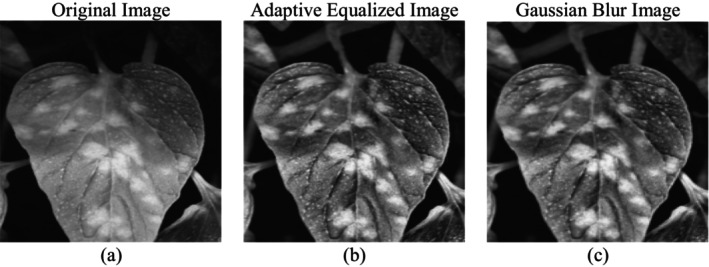
Dataset preprocessing (a) original image, (b) adaptive equalized image, and (c) Gaussian blur image.

Figure 3 shows the preprocessing operations performed on the plant leaf images for the segmentation of disease and training of deep‐learning models. It comprises three images: (a) original, (b) adaptive equalized, and (c) Gaussian filtered, each depicting a various phase of preprocessing. The raw input grayscale Figure 3a is the original image. The original image may contain variations in light, contrast, and noise. Variations in image quality can make it difficult for a segmentation model to differentiate diseased and healthy regions accurately. Shadows and uneven brightness can conceal vital features on which the effective detection of disease depends. The equalized adaptive Figure 3b is the result of adaptive histogram equalization, an algorithm utilized for contrast improvement using redistribution of pixel intensity levels. Adaptive equalization increases the local contrast in the different parts of the image such that diseased areas are rendered more apparent. Adaptive equalization helps to highlight small disease marks that would not be noticed otherwise by normalizing brightness differences. The Gaussian‐filtered Figure 3c is derived through a Gaussian blur, which smooths the image to eliminate noise but preserves edges that are necessary. The filtering operation eliminates extraneous variation caused by external factors such as non‐uniform illumination or environmental noise from the scene so that the model of segmentation is able to focus on significant patterns of disease. The application of the Gaussian filter keeps only crucial structures salient and makes readability better in the diseased area.

### 3.3 Data Augmentation

The range of data augmentation techniques has been applied to the training images to enhance model generalization and reduce overfitting. Five distinct augmentation types were used to simulate real‐world variations commonly encountered in field conditions, such as changes in orientation, brightness, and leaf deformation. Table 1 outlines the data augmentation techniques applied to the training set to improve model robustness and generalization. Horizontal and vertical flips were applied randomly with a probability of 0.5 each to teach the model orientation‐invariant features. Random rotation within ±20° helped the model learn to segment leaves from various angles. Brightness adjustments within a ±20% range were implemented to mimic varying lighting conditions. Finally, elastic transformations using *α* = 34 and *σ* = 4 (via the Albumentations library) introduced slight warping in the images, replicating natural leaf deformation. These transformations collectively enriched the diversity of the training data without altering the semantic content, thereby enhancing the model's ability to accurately detect diseased regions under diverse imaging scenarios.

**TABLE 1:** Data augmentation parameters.

Augmentation type	Description	Parameter
Horizontal flip	Random horizontal mirroring	Probability = 0.5
Vertical flip	Random vertical mirroring	Probability = 0.5
Rotation	Random rotation of the image	Angle range = ±20°
Brightness adjustment	Adjusts the brightness	Factor range = ±20%
Elastic transformation	Warps the image to simulate deformation	*α* = 34, *σ* = 4 (Albumentations)

**FIGURE 4:**
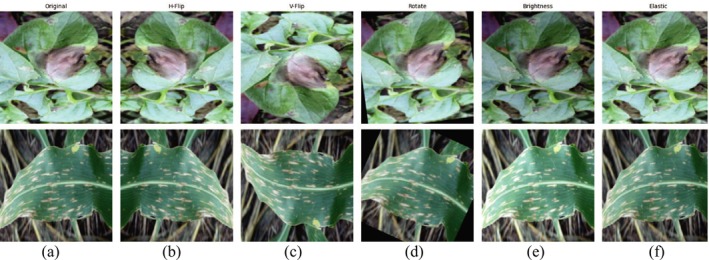
Data augmentation (a) original image, (b) horizontal flip, (c) vertical flip, (d) rotate, (e) brightness, and (f) elastic.

The original image, shown in Figure 4a, serves as the baseline, while the augmented images demonstrate the applied transformations. A horizontal flip in Figure 4b and a vertical flip in Figure 4c were applied with a probability of 0.5 each, enabling the model to learn orientation‐invariant features. Rotation in Figure 4d was performed randomly within a range of ±20°, simulating natural variations in leaf orientation during image acquisition. Brightness adjustment in Figure 4e was implemented with a variation factor of ±20% to mimic differing lighting conditions commonly encountered in agricultural environments. Lastly, an elastic transformation in Figure 4f was applied with an alpha value of 34 and a sigma of 4, introducing small spatial distortions to replicate natural bending or warping of leaves. These augmentation techniques were implemented using the Albumentations library and were intended to diversify the dataset, thereby enhancing the segmentation model's robustness and reliability across a range of visual conditions.

### 3.4 Dataset Splitting

Table 2 presents the distribution of the dataset across training, validation, and testing subsets before and after augmentation. Out of the total 586 original images, 470 were used for training, while 58 each were allocated to validation and testing. Data augmentation was applied exclusively to the training set to increase data diversity and reduce overfitting. A total of five augmentation techniques (horizontal flip, vertical flip, rotation, brightness adjustment, and elastic transformation) were applied, generating five additional variants per original training image, resulting in 2350 augmented samples. This brought the total number of training images to 2820, while the validation and testing sets remained unchanged to ensure unbiased performance evaluation. The final dataset used in training and evaluation consisted of 2936 images, significantly enhancing the model's exposure to visual diversity without altering the original ground‐truth integrity.

**TABLE 2:** Dataset splitting.

Subset	Original images	Number of augmentations	Augmented images (×5)	Total images (original + augmented)
Training	470	5	2350	2820
Validation	58	0	0	58
Testing	58	0	0	58
Total	586	—	2350	2936

### 3.5 Proposed DBA‐DeepLab Model

**FIGURE 5:**
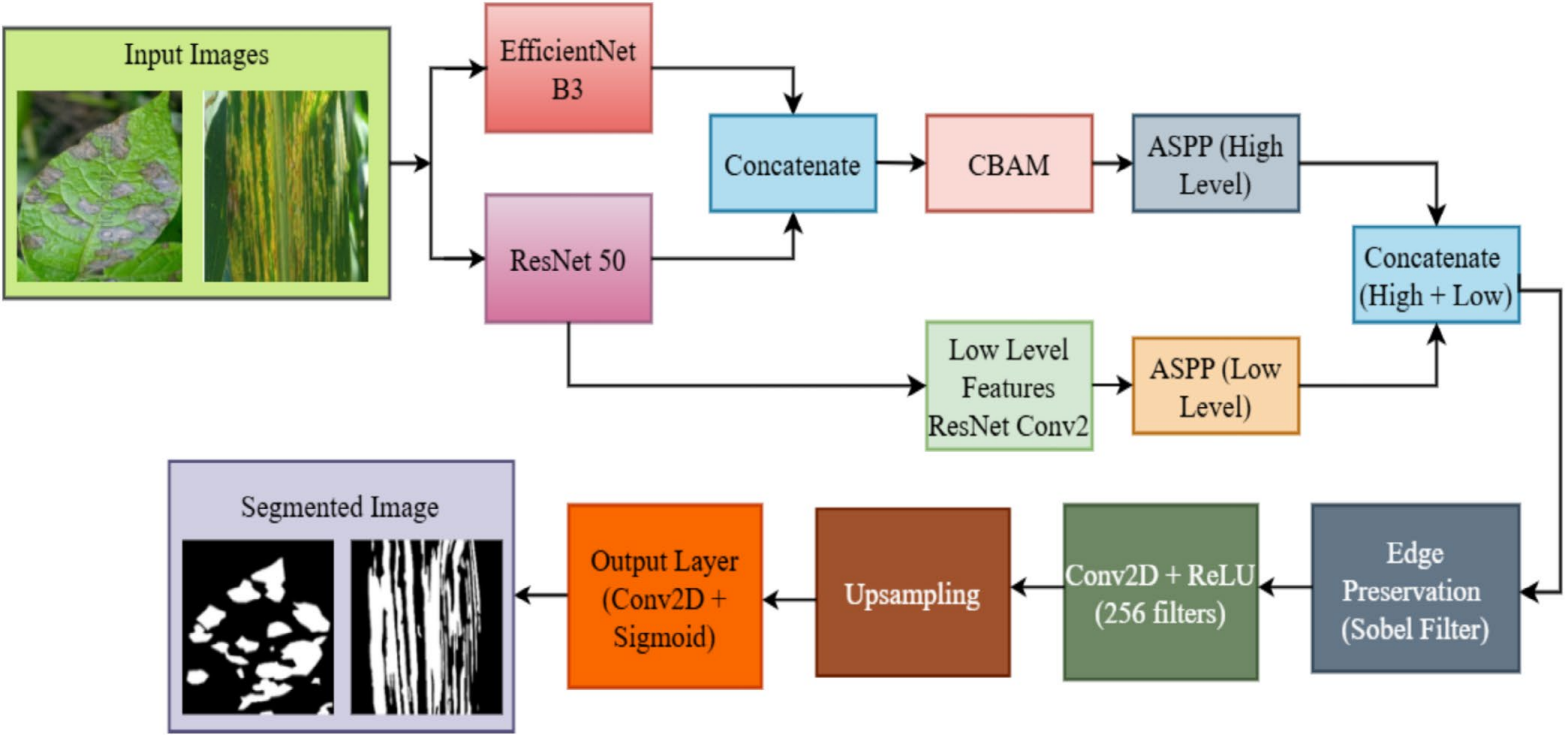
Proposed DBA–DeepLab V3+ model.

The proposed DBA‐DeepLab model is DeepLabV3+, which has been boosted through a dual‐backbone network, attention (CBAM), and an edge detection module (Sobel filter) to better enhance plant leaf disease segmentation. Figure 5 provides a description of the proposed architecture of the DeepLabV3+ model utilized in plant disease segmentation using a dual‐backbone network, attention models, multi‐scale feature extraction, and edge retention processes. The model adopts a methodical process with raw input images as the input and goes through several layers of processing to yield highly precise segmentation masks. The architecture is particularly designed to improve disease localization accuracy on plant leaves, despite the common weaknesses of changes in lighting, complicated backgrounds, and variability in the severity of the diseases.

The segmentation pipeline begins from the input images, such as leaves of a plant with obvious indications of diseases like discoloration, lesions, and necrotic spots. The images are passed through a two‐backbone feature extraction process of EfficientNet‐B3 and ResNet‐50, which are two deep‐learning models with sufficient capability that function together to improve feature representation. EfficientNet‐B3 is employed due to its light and scalable structure with depth, width, and resolution optimized for optimal performance. It assists in ensuring the network learns significant high‐level contextual information effectively in computations, maintaining low complexity while accuracy of segmentations is retained. ResNet‐50, due to its residual learning capability, is used to ensure fine‐grained details are retained and feature extraction is boosted in deeper layers. This assists in picking up minute variations in the symptoms of the disease that are invisible against the backdrop. Upon feature extraction from each of the two backbones, both are fused subsequently, thereby enabling the model to capitalize on the best aspects of the two networks. Fusing the EfficientNet‐B3 scale‐optimized and spatial ResNet‐50 high‐resolution features provides a more robust and extensive feature description, which is critical for successful segmentation of disease areas from the area of healthy leaves. Following feature extraction, the model utilizes the CBAM for additional feature selection and disease localization. CBAM is a joint spatial and channel attention mechanism. The latter dynamically scales down the model's attention to highlight the most relevant portions of the image and discard irrelevant background features. Spatial attention assists the model in highlighting disease areas by considering positional information, while channel attention discards unwanted features and promotes the most important features with respect to disease patterns. The incorporation of CBAM greatly enhances the segmentation accuracy by having the network focus on disease‐subsistent regions instead of being distracted by areas of the image that are not related. The second important step in the architecture is ASPP, and this is separately applied to both high‐level and low‐level feature maps. High‐level ASPP is applied to concatenated features of the dual‐backbone network, and it captures multi‐scale contextual information. This is necessary for segmenting disease areas of different sizes and shapes since plant diseases can manifest as small localized lesions or cover extensive areas of the leaf. Low‐level ASPP, when applied to early convolutional layers of ResNet‐50, preserves fine spatial information so that even the smallest disease areas are correctly identified. The outputs of the low‐ and high‐level ASPP modules are then concatenated, enabling the model to bring together both global contextual information and fine‐grained local features for more accurate segmentation. For additional boundary precision enhancement, the edge preservation module is suggested based on the Sobel filter, a widely used edge detection technique that highlights sudden intensity changes in images.

This stage ensures that the model properly delineates the edges between diseased and healthy regions, improving segmentation quality and reducing false positives and negatives. The result is then convolved with a 256‐filter convolutional layer with ReLU activation, sharpening the extracted edge features prior to final upsampling. Finally, the upsampling layer in the next step upsamples the segmented output into the same resolution as the input image so that the resultant segmentation mask remains properly aligned with the input image. This step is important to offer spatial coherence, and the processed feature maps well map the diseased areas to their appropriate locations in the plant leaf. Finally, the feature maps that have gone through processing are sent to the output layer, which is Conv2D with the sigmoid function. The output is a binary segmentation mask where white means diseased areas and black means healthy tissues or background. The result gives an easy and clean segmentation of the infected regions, and this can be useful in autonomous plant disease inspection and precision agriculture. The DeepLabV3+ model proposed here far surpasses current segmentation models like DeepLab V3+ with EfficientNet B3, DeepLab V3+ with ResNet 50, and DeepLab V3 with Dual Backbone because it includes dual backbones, CBAM for attention‐based refinement, ASPP for multi‐scale learning of features, and Sobel filtering for accurate edge preservation. These updates together allow the model to produce higher accuracy, better IoU (intersection over union) scores, and better Dice coefficient scores, becoming more accurate for real‐world agricultural use. This model is especially useful for precision agriculture and automatic plant disease diagnosis since it allows for early intervention through highly precise segmentation of the infected areas. By combining cutting‐edge deep learning methods with feature extraction, attention mechanisms, multi‐scale context learning, and edge detection, the model is an efficient and effective solution for future smart agriculture and plant disease studies.

#### 3.5.1 Feature Extraction From EfficientNet B3 Backbone

EfficientNet‐B3 is employed as a backbone network in the DeepLabV3+ model proposed for effective and accurate feature extraction of plant disease segmentation. In contrast to conventional deep networks, EfficientNet‐B3 employs compound scaling, which maintains depth, width, and resolution in the network in an optimal manner to produce the optimal performance at the least computational expense. This guarantees the network learns spatial information efficiently. Mobile inverted bottleneck convolution (MBConv) blocks employed in EfficientNet‐B3 also enhance feature extraction via depthwise separable convolutions by removing unnecessary computation without losing important structural details. These aspects render EfficientNet‐B3 highly adaptable to segmentation tasks, where it is absolutely necessary to identify even minor differences between diseased and normal plant regions.

Second, EfficientNet‐B3 comes with a squeeze‐and‐excitation (SE) module, permitting the relative weights of various channels to change dynamically, which facilitates it to easily concentrate on areas of disease with ease and rid itself of excessive background information. This is vital in plant disease segmentation, in which symptoms such as discoloration, necrotic spots, or lesions also differ in intensity and magnitude. Through the integration of EfficientNet‐B3 and ResNet‐50 within a dual‐backbone framework, the new model enjoys compact and high‐resolution feature extraction (EfficientNet‐B3) and deep contextual learning (ResNet‐50). With such a combination, segmentation precision is enhanced, false positives are reduced, and the localization of disease areas is also improved. The efficient structure of EfficientNet‐B3 also brings about shorter training and inference speeds, which results in a very practical solution for the real‐time detection of plant disease in smart agricultural applications.

**FIGURE 6:**
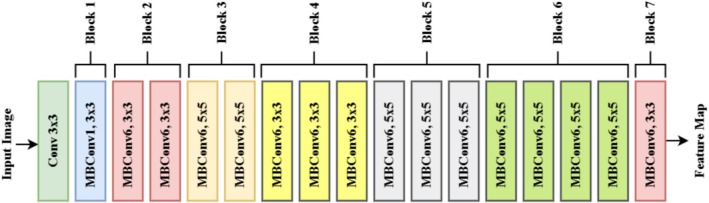
Architecture of EfficientNet B3 model.

Figure 6 depicts the architecture of a CNN backbone, which is particularly inspired by the EfficientNet (Chowdhury et al. 2020) architecture that uses MBConv layers for feature extraction. The model is organized into several blocks, each of which contains various convolutional layers used to extract hierarchical features from an input image. The process starts with the initial 3 × 3 convolutional layer, which has the role of detecting simple edge and texture details of the input image. The MBConv layers follow, which are depthwise separable convolutions that enhance computational efficiency without affecting high accuracy. All of these seven blocks apply different expansion rates and kernel sizes to enhance feature representation. Block 1 involves an MBConv layer with a 3 × 3 kernel that assists in extracting local spatial information at a low computational cost. Block 2 and Block 3 add MBConv6 layers using both 3 × 3 and 5 × 5 kernels, where the expansion factor (6×) enables the model to learn richer representations through expanding the channels before performing depthwise convolution. The usage of 5 × 5 kernels during later stages helps the model handle bigger receptive fields, which are needed to extract more abstract features. As the network goes from Blocks 4 through 6, additional MBConv6 with 3 × 3 and 5 × 5 kernels are applied, further elaborating the feature maps by pulling out mid‐level and high‐level representations. These layers are responsible for learning parts of the learning object, texture, and patterns, which are essential for classification and segmentation. The repetition of MBConv6 layers ensures efficiency while learning deep hierarchical structures in the input data. Lastly, Block 7 contains the last MBConv6 with a 3 × 3 kernel to produce the feature map output, which is the previous set of extracted features employed for further operations, i.e., classification, object detection, or segmentation. The architecture achieves an effective trade‐off in depth, width, and resolution while keeping the model lightweight and very efficient in feature extraction. The application of MBConv layers in various configurations assists in learning fine‐grained and large‐scale features, thus making this backbone suitable for image classification, object detection, and segmentation tasks in deep learning applications.

**FIGURE 7:**
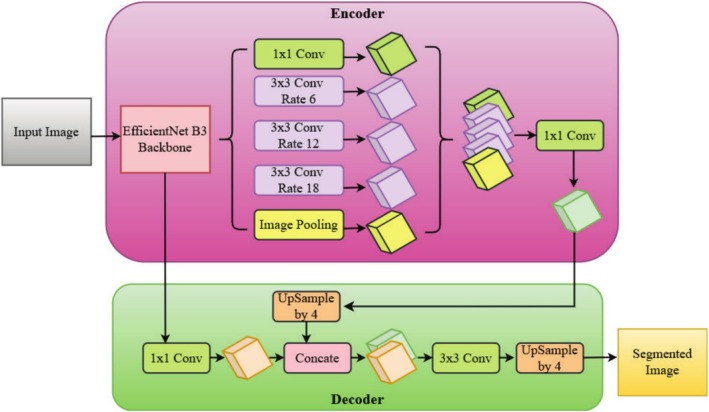
DeepLab V3+ with EfficientNet B3 backbone.

DeepLabV3+ with EfficientNet‐B3 as backbone architecture is illustrated in Figure 7, with emphasis on its encoder‐decoder semantic segmentation architecture. The pipeline begins from an input image, which is inputted to the EfficientNet‐B3 backbone, an extremely optimized and light convolutional neural network for feature extraction in an efficient manner. EfficientNet‐B3 incorporates depthwise separable convolutions and mobile inverted bottleneck blocks (MBConv), thus enabling the network to simultaneously capture fine‐grained spatial patterns as well as contextual high‐level information while maintaining efficiency in computations. Through this approach, the network is able to detect areas affected by diseases in leaves of plants effectively. Feature extraction is multi‐scale in application in the encoder module with the ASPP. The feature maps extracted are subjected to 1 × 1 convolution to address low‐level details and three parallel 3 × 3 convolutions with varying dilation rates (6, 12, and 18). The dilation rates enable the network to detect disease areas of different sizes and intensities, hence making it more appropriate to segment lesions and other symptoms accurately. Furthermore, image pooling is utilized to pool global contextual information to allow the model to learn both local textures and global structural patterns. The output of these convolutions is concatenated and passed through another 1 × 1 convolution for additional fine‐grained multi‐scale feature extraction.

The decoder module restores the segmentation output by iteratively upsampling the encoded feature maps. A 1 × 1 convolution is first utilized to restore fine details and then concatenated with low‐level features from the shallow layers of EfficientNet‐B3. Refinement of the concatenated features is accomplished using a 3 × 3 convolution to enhance edge accuracy and boundary sharpness. Lastly, two consecutive upsampling steps (by a factor of 4) are applied to recover the original image resolution and generate a high‐quality segmentation mask where diseased areas are properly emphasized. This architecture successfully strikes a balance between efficiency and segmentation performance by taking advantage of EfficientNet‐B3's lightweight but strong feature extraction and DeepLabV3+'s multi‐scale learning. The combination of ASPP, feature refinement, and progressive upsampling guarantees that the model can effectively identify plant diseases at various scales and backgrounds and thus is extremely appropriate for precision agriculture and automatic disease monitoring systems.

#### 3.5.2 Feature Extraction From ResNet 50 Backbone

ResNet‐50 is used as a basic backbone in the DeepLabV3+ model proposed here, offering deep hierarchical feature extraction for accurate plant disease segmentation. ResNet‐50 is widely recognized for its residual learning mechanism, which successfully avoids the vanishing gradient issue, enabling deeper network training without performance loss. Through skip connections, ResNet‐50 preserves low‐level spatial information while, at the same time, extracting high‐level semantic information. This property is critical for segmenting plant disease regions, where both fine‐grained textural details and large‐scale structural variations need to be identified. Additionally, the residual blocks allow the network to identify subtle leaf disease patterns, improving the discrimination between diseased and healthy regions, even in challenging backgrounds.

The bottleneck architecture of ResNet‐50 also enhances computation efficiency through the use of 1 × 1 convolutions in reducing feature map sizes before using 3 × 3 convolutions without impairing accuracy and optimizing memory utilization. This feature is required to handle high‐resolution images of plants, where correct segmentation is imperative. In the proposed dual‐backbone model, ResNet‐50 improves EfficientNet‐B3 by focusing on deeper feature representations to enhance the model's performance in segmenting diseased regions with varying sizes, shapes, and intensity values. By fusing ResNet‐50's deep feature extraction capability with EfficientNet‐B3's light feature representation capability, the proposed model improves segmentation accuracy and disease symptom localization and reduces misclassification errors. This makes ResNet‐50 an integral component of the proposed architecture and contributes significantly to its robust performance in plant disease detection and precision agriculture tasks.

**FIGURE 8:**
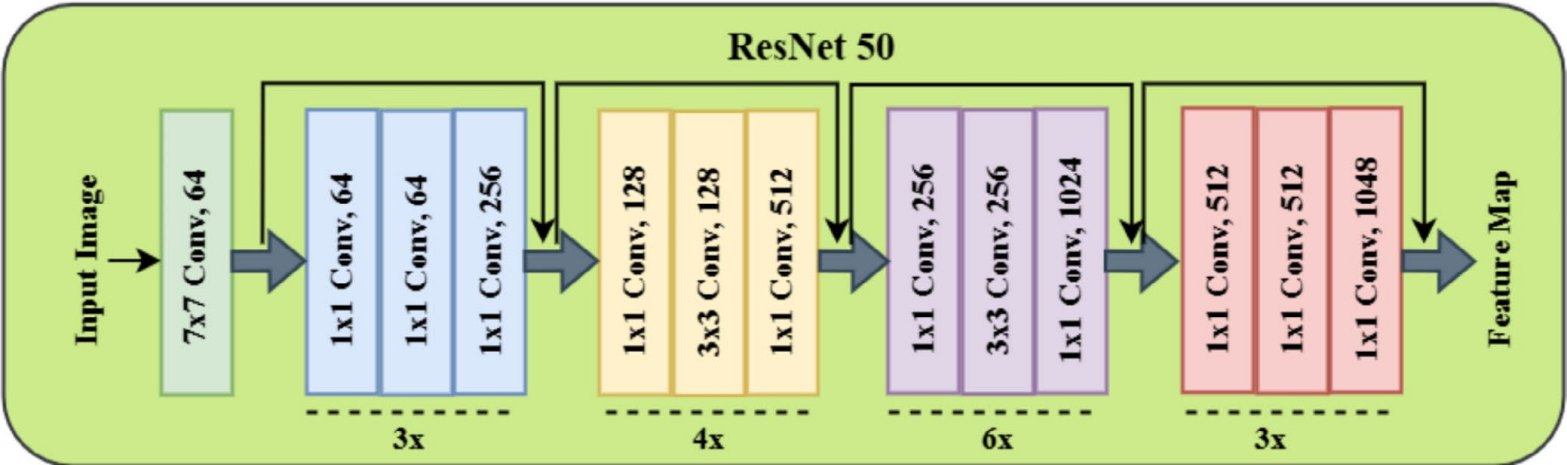
Architecture of ResNet 50 model.

ResNet‐50 architecture (Koonce and Koonce 2021), a deep CNN widely used for feature extraction in deep learning, is depicted in Figure 8. It includes a series of convolutional layers with residual blocks, which help the networks train effectively by eliminating the vanishing gradient problem. The architecture has a structured sequence, starting from an input image and passing through various convolutional and residual layers until arriving at a final feature map. At the end, the ResNet‐50 architecture is displayed, demonstrating the hierarchical structure of the convolutional layers. The process is initiated by a 7 × 7 convolutional layer with 64 filters that extract low‐level spatial features such as edges and textures. This is then followed by a series of residual blocks, with each containing multiple convolutional layers. The first residual block contains 1 × 1, 3 × 3, and 1 × 1 convolutional layers, allowing for efficient feature extraction while maintaining computational efficiency (Cai et al. 2022). These layers increase in‐depth gradually, from 64 filters in earlier layers to 1048 filters in the last stage, to ensure deep hierarchical features are extracted (Zhang et al. 2021). The upper part of Figure 7 indicates the residual block architecture, which is the central unit of ResNet‐50.

In each residual block, input features (represented as X) go through a sequence of weight layers with ReLU activation, resulting in an output representation (F(X)). Rather than directly feeding the transformed features to the subsequent layer, ResNet employs a skip connection, adding the input X to the transformed F(X). The process, residual learning, maintains critical information within the network and avoids gradient vanishing in deep networks. Following summation (F(X) + X), another ReLU activation is used to add non‐linearity and enhance feature learning. Applying 1 × 1 convolutions in every residual block has a variety of functionalities, such as channel‐wise feature enhancement and dimensionality reduction, while applying 3 × 3 convolutions extracts spatial patterns. Increasing the number of filters as the network goes deeper enables deeper layers to analyze more complex and abstract representations. The hierarchical architecture of ResNet‐50 makes it highly efficient for object detection, segmentation, and image classification. Overall, the architecture is capable of deep feature extraction while preserving computational efficiency and smooth gradient flow. By combining skip connections, residual learning, and deep convolutional layers, ResNet‐50 achieves high accuracy in many computer vision tasks. It is a widely accepted backbone model for deep learning‐based feature extraction.

**FIGURE 9:**
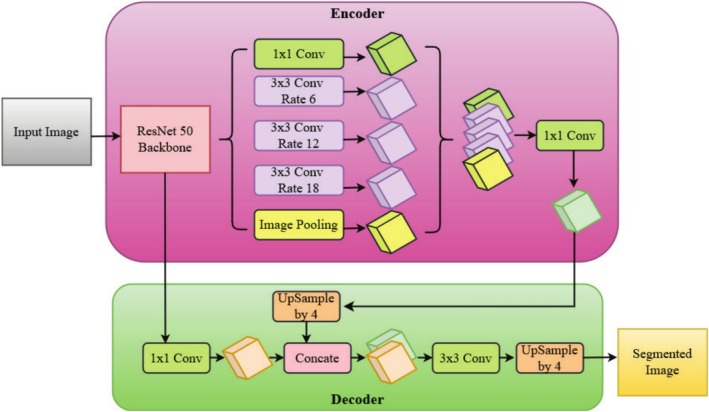
DeepLab V3+ with ResNet 50 backbone.

The DeepLabV3+ architecture with ResNet‐50 as the backbone is illustrated in Figure 9, showing its encoder‐decoder structure for high‐precision semantic segmentation. Segmentation begins with an input image, which passes through the ResNet‐50 backbone, a deep convolutional neural network that specializes in hierarchical feature extraction. ResNet‐50 employs residual learning through skip connections, which maintain spatial information and accurately extract low‐level and high‐level features. This enables the model to capture meaningful representations of disease regions in plant leaves without instability during deep network training. The encoder module is made up of ASPP, which extracts multi‐scale features by using dilated convolutions with various rates (6, 12, and 18). The convolutions enable the network to identify disease lesions of different sizes and intensities without sacrificing spatial resolution. Further, image pooling is carried out to combine global contextual information so that the network learns fine details and global structural patterns within the diseased areas. Feature maps are fine‐tuned by applying a 1 × 1 convolution at various stages prior to concatenation and maintaining fine‐grained spatial information.

The decoder module re‐establishes the segmentation output by upscaling the obtained features to be equivalent to the original input image resolution. First, a 1 × 1 convolution is used to fine‐tune high‐level feature representations prior to concatenation with low‐level features from previous layers of ResNet‐50. This preserves the fine edge details in the segmentation model and enhances boundary detection of diseased regions. Next, a 3 × 3 convolution is used to refine features further, followed by two successive upsampling steps (each by a factor of 4) to recover the original spatial resolution. The resulting segmentation mask is the final segmentation mask, and the diseased regions are accurately segmented from the normal plant tissue. This DeepLabV3+ with a ResNet‐50 backbone achieves a good balance between depth and feature learning and is thus a robust model for plant disease segmentation. With residual learning, ASPP for multi‐scale context learning, and an efficient decoder, this architecture is able to achieve good segmentation accuracy at low computational cost. Its ability to identify fine details as well as large‐scale patterns makes it highly suitable for precision agriculture and automated plant disease detection systems.

#### 3.5.3 Concatenation of Features From Dual Backbone

**FIGURE 10:**
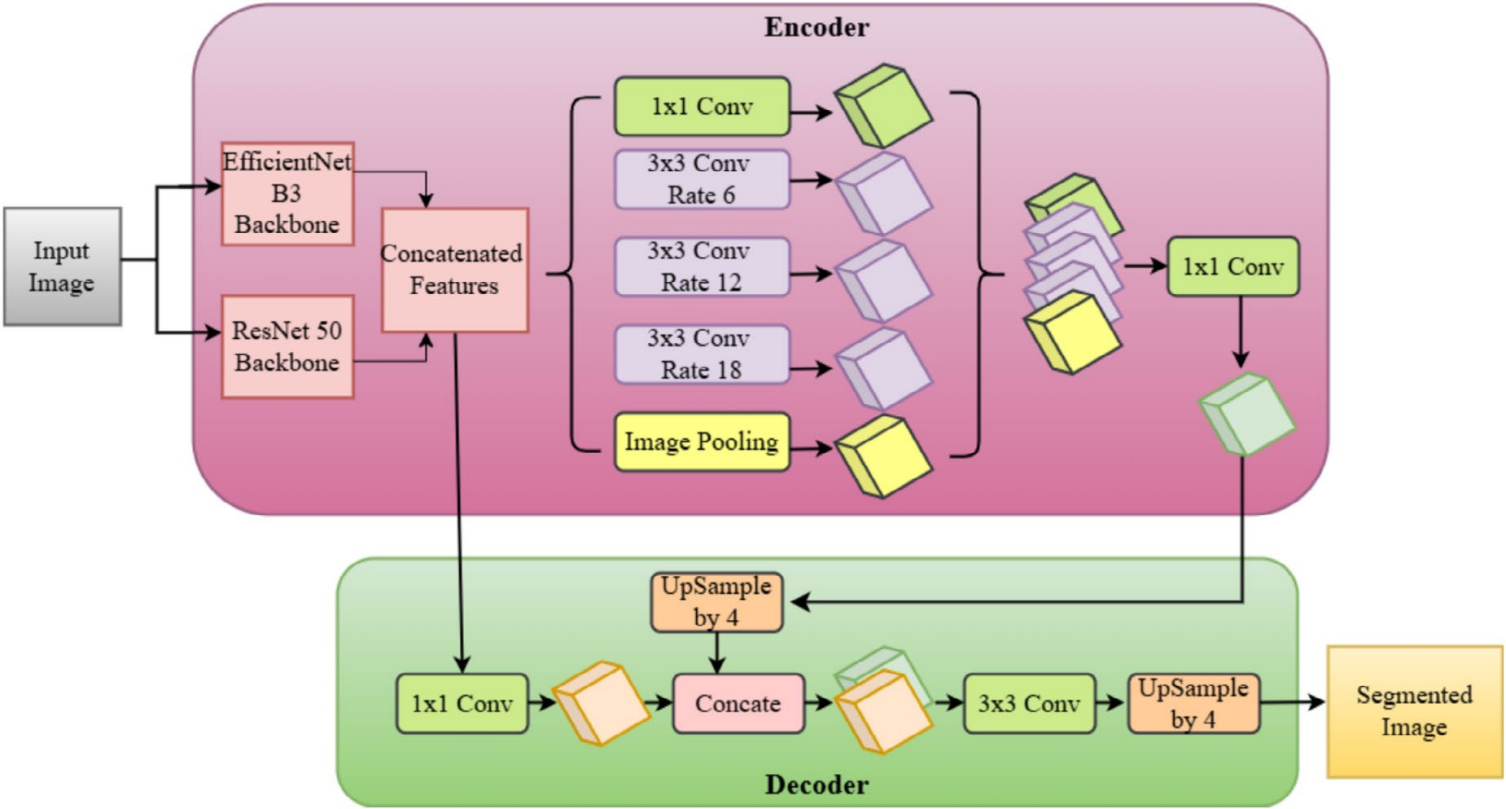
DeepLab V3+ with dual backbone (EfficientNet B3 and ResNet 50).

The dual‐backbone architecture of DeepLabV3+ is presented in Figure 10, where EfficientNet‐B3 and ResNet‐50 are used together for enhanced plant disease segmentation. The hybrid feature extraction approach leverages the strengths of both backbones while balancing deep contextual feature learning and computational cost. The pipeline begins with an input image, which is passed through EfficientNet‐B3 and ResNet‐50 individually. EfficientNet‐B3 imparts feature engineering optimized with its compound scaling methodology to maintain effective depth, width, and resolution management, and ResNet‐50, owing to its residual learning architecture, is able to retain deep hierarchical features essential for accurate segmentation.

Feature maps retrieved from both backbone models are stacked together so the model can integrate high‐resolution spatial information from EfficientNet‐B3 and rich semantic knowledge from ResNet‐50. This combination yields a strong feature representation, which proves to be extremely effective in detecting disease‐infected areas on the leaves of plants. The merged features are thereafter fed into the encoder module, and ASPP is performed. ASPP includes several 3 × 3 convolutional layers with various dilation rates (6, 12, and 18) to extract multi‐scale contextual information so that the model can effectively identify disease lesions of different sizes and intensities. Image pooling is also added to preserve global contextual features, further improving the segmentation performance. In the decoder module, the high‐level features extracted are refined by a 1 × 1 convolution before progressive upsampling. The decoder combines these fine features with low‐level spatial information from the previous layers to achieve precise boundary detection and segmentation. The feature refinement is additionally boosted by a 3 × 3 convolution before the ultimate upsampling operations (by a factor of 4) return the spatial resolution to the original input size. The ultimate segmentation mask delineates disease‐affected areas with high accuracy, separating them from normal plant tissues. This two‐backbone DeepLabV3+ architecture greatly improves segmentation precision and resilience over conventional single‐backbone architectures. Through the synergy of EfficientNet‐B3's efficiency with ResNet‐50's deep feature extraction ability, the network provides high‐performance results in precision agriculture and autonomous plant disease monitoring, which can be a great tool for real‐world applications of smart farming.

#### 3.5.4 Integration of CBAM

**FIGURE 11:**
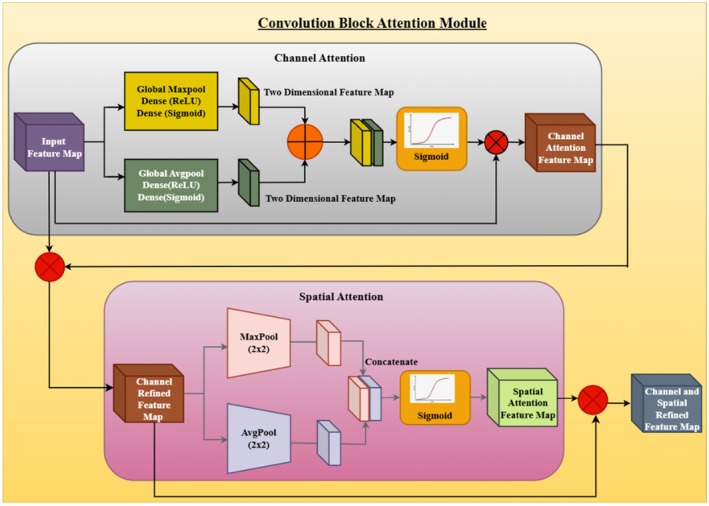
Convolutional block attention module (CBAM).

Figure 11 shows the CBAM, an attention mechanism employed to strengthen feature representation in deep learning algorithms by using channel attention and spatial attention sequentially.

CBAM augments the ability of CNNs to focus on informative features while suppressing redundant information. Therefore, it is particularly useful in areas such as image segmentation, object detection, and classification. The architecture begins from an input feature map and passes through two major attention modules: channel attention and spatial attention. The two modules collaborate to fine‐tune feature maps such that the network can effectively learn the most informative areas of the input image. The channel attention module operates by fine‐tuning feature maps to strengthen the most relevant channels and weaken the effects of the less relevant channels. These operations are global max pooling and global average pooling, both applied along the spatial axes of the input feature map. These are fed into dense layers with ReLU activation and followed by a sigmoid activation function to produce a two‐dimensional feature map describing the relative weight of every channel. The results of max pooling and mean pooling are added via element‐wise multiplication to get the channel attention feature map, which is then passed to the next step. Following attention refinement at the channel level, spatial attention refines feature representation even more by picking the most critical spatial locations out of the feature maps. Max pooling and average pooling operations are carried out on the channel‐refined feature map across the channel axis. The output of the two pooling operations is concatenated and passed through a sigmoid activation function to yield a spatial attention feature map that highlights important spatial regions in the image. This refined spatial attention map is then multiplied element‐wise with the input feature map to produce the final channel and spatial refined feature map. Channel attention and spatial attention are used together to ensure that the final feature map contains only the most vital information and removes redundant or less vital details. This improved feature representation improves the performance of deep‐learning models in a variety of tasks through better localization and discrimination of the most important image regions. Overall, CBAM offers a light and efficient attention mechanism that greatly improves feature extraction ability in CNNs. By integrating global contextual information with channel attention and local spatial information with spatial attention, CBAM enables more accurate feature learning and deep learning‐based vision applications.

#### 3.5.5 Integration of ASPP

**FIGURE 12:**
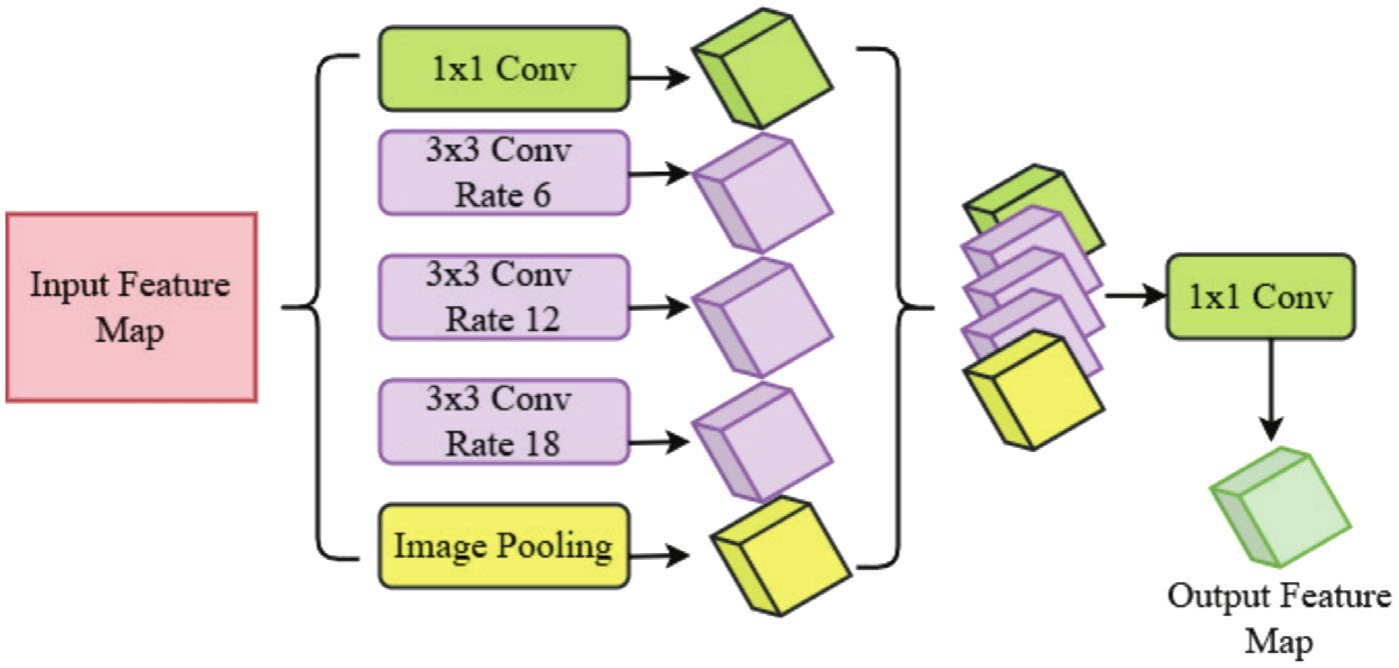
Atrous spatial pyramid pooling module.

Figure 12 is an ASPP block, which is a core component of deep learning‐based semantic segmentation models like DeepLabV3+. The ASPP block tries to learn multi‐scale context features by applying a sequence of convolutional operations with different dilation rates, having an expanded receptive field but retaining the fine spatial information. This ability to pull data from different scales helps the model detect objects of varying sizes and shapes more effectively. The process starts with an input feature map, which goes through a series of parallel transformations to extract local fine details along with global context data. The first is a 1 × 1 convolution, applied to reduce the dimensionality of the input while maintaining spatial resolution. Thereafter, several 3 × 3 convolutions with different dilation rates are used, where dilation rates of 6, 12, and 18 expand the receptive field progressively without the corresponding increase in computational cost. The lower dilation rate picks up mid‐level details, and the higher dilation rates pick up larger structural patterns across the image. These dilated convolutions enable the model to understand fine‐grained textures as well as large‐scale contextual relationships. Further, global average pooling is used to condense the whole image into one feature representation, retaining the most prominent contextual information. The pooling operation aids in the identification of large‐scale patterns that may not be possible with single convolutions. Subsequent to these operations, the feature maps that are extracted from various branches are concatenated so that the model can combine features across multiple scales. The concatenated result is fed to a last 1 × 1 convolutional layer, where it refines the combined features to produce the output feature map.

The ASPP module also serves an important function in enhancing the accuracy of segmentation since it makes the model scale‐invariant and, therefore, can effectively detect objects and regions of varying sizes. Through the use of multi‐scale information, ASPP boosts the capability of CNN‐based segmentation models to delineate background from foreground regions. Hence, it is very effective for plant disease segmentation, medical imaging, and urban scene understanding. This module greatly enhances the strength and accuracy of deep‐learning models, making them more effective for real‐world usage where segmentation precision is paramount.

### 3.6 Mathematical Representation of the Proposed DBA‐DeepLab Model

The input image I is a high‐resolution RGB image with height *H*, width *W*, and three channels (RGB). This input is normalized and preprocessed using mean subtraction and scaling as shown in Equation (1)

(1) $$I^{\prime }=\frac{I-\mu }{\sigma }$$

The features of the input image are extracted using ResNet 50 and EfficientNet B3 as shown in Equations (2) and (3)

(2) $$F_{\text{ResNet}}=\text{ResNet}50\left(I^{\prime }\right)$$

(3) $$F_{\text{EffNet}}=\text{EfficientNetB}3\left(I^{\prime }\right)$$

The extracted features of Equation (2) and (3) are concatenated as shown in Equation (4)

(4) $$F_{\text{dual}}=\text{Concat}\left(F_{\text{ResNet}},F_{\text{EffNet}}\right)$$

After the feature extraction using dual backbones, the Attention Mechanism is applied on the images using the CBAM Module. Firstly, channel attention is applied, whose output is shown in the Equation in (5)

(5) $$M_{c}=\sigma \left(W_{2}\text{ReLU}\left(W_{1}\left(\text{AvgPool}\left(F_{\text{dual}}\right)+\text{MaxPool}\left(F_{\text{dual}}\right)\right)\right)\right)$$

The refined feature map after channel attention is shown in Equation (6)

(6) $$F_{c}=M_{c}\cdot F_{\text{dual}}$$

After channel attention, the spatial attention is applied as shown in Equation (7)

(7) $$M_{s}=\sigma \left({\text{Conv}}_{7\times 7}\left(\left[\text{AvgPool}\left(F_{c}\right),\text{MaxPool}\left(F_{c}\right)\right]\right)\right)$$

The concatenated feature map after the attention mechanism is as shown in Equation (8)

(8) $$F_{\text{CBAM}}=M_{s}\cdot F_{c}$$

After attention refinement, the feature map undergoes ASPP for multi‐scale context learning as shown in Equation (9)

$$F_{\text{ASPP}}=\text{Concat}({\text{Conv}}_{1\times 1}\left(F_{\text{CBAM}}\right)$$

$${\text{Conv}}_{3\times 3,\;\text{rate}=6}\left(F_{\text{CBAM}}\right)$$

$${\text{Conv}}_{3\times 3,\text{rate}=12}\left(F_{\text{CBAM}}\right),{\text{Conv}}_{3\times 3,\text{rate}=18}\left(F_{\text{CBAM}}\right)$$

(9) $$\text{GlobalPool}\left(F_{\text{CBAM}}\right))$$

The output from ASPP is upsampled to match the input resolution as shown in Equation (10)

(10) $$F_{\text{Upsample}}=\text{Upsample}\;\left(F_{\text{ASPP}}\right)$$

Finally, the segmentation mask is generated using a 1 × 1 convolution and sigmoid activation as shown in Equation (11)

(11) $$\text{Mask}=\sigma \left({\text{Conv}}_{1\times 1}\left(F_{\text{Upsample}}\right)\right)$$

Where Mask is the final binary segmentation map of the proposed DBA‐DeepLab model.

## 4 Results and Discussion

The proposed DBA‐DeepLab model with dual backbones (ResNet‐50 and EfficientNet‐B3) and the CBAM attention module was trained and evaluated for plant disease segmentation using a well‐defined simulation environment. The model was implemented in Google Colab utilizing a GPU‐accelerated environment, which significantly enhanced computational efficiency and reduced training time. The deep learning framework used for model development and training was TensorFlow and Keras, which provided robust tools for designing, training, and fine‐tuning the segmentation network. The model was trained using a learning rate of 0.0001, which ensured stable convergence and prevented the network from overshooting the optimal solution. A batch size of 8 was used to balance memory efficiency and model performance, allowing effective mini‐batch gradient updates. The optimization of the model was performed using the Adam optimizer, known for its adaptive learning rate adjustments and efficient handling of sparse gradients. The network was trained for 50 epochs, ensuring sufficient iterations for model convergence while avoiding overfitting. The following results demonstrate significant improvements in segmentation accuracy, loss reduction, and boundary delineation compared to DeepLabV3+ with ResNet 50, DeepLabV3+ with EfficientNet B3, DeepLabV3+ with Dual Backbone, and the proposed DBA‐DeepLab model.

### 4.1 Results of DeepLab V3+ With EfficientNet B3 Backbone

**FIGURE 13:**
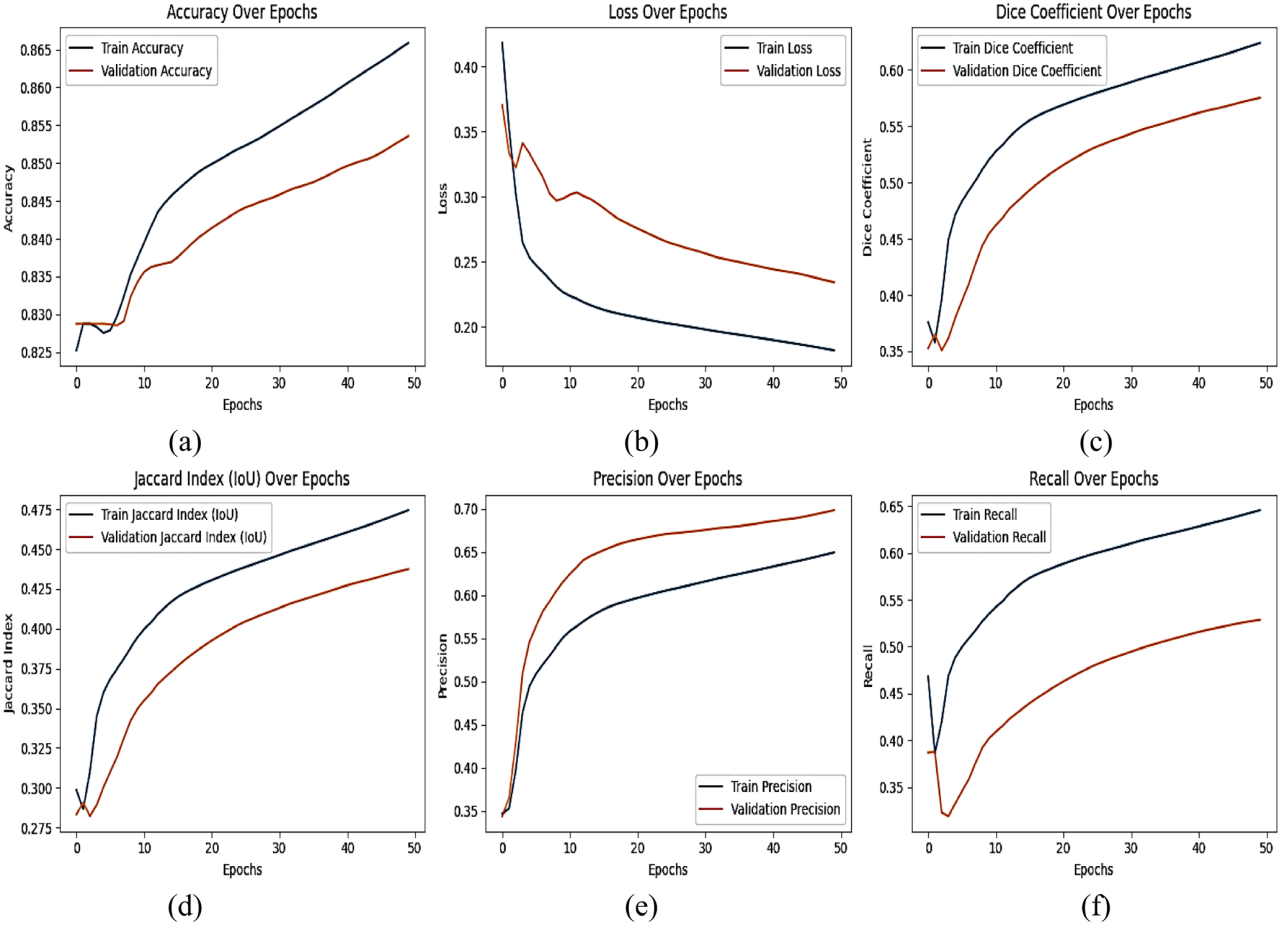
Training and validation curve using DeepLab V3+ with EfficientNet B3 model (a) accuracy, (b) loss, (c) Dice coefficient, (d) Jaccard index, (e) precision, and (f) recall.

Figure 13 shows the revised training and validation performance curves of the DeepLabV3+ model with the EfficientNet‐B3 backbone across 50 epochs. The performance is measured by six of the most important segmentation metrics: accuracy, loss, Dice coefficient, Jaccard Index (IoU), precision, and recall. The plots demonstrate the learning dynamics of the model, its generalization capability, and its segmentation effectiveness. Figure 13a illustrates the training and validation accuracy curves depicting a smooth increase across epochs, meaning steady learning progress. The model begins with low accuracy during early epochs but enhances consistently to approximately 0.865 for training and 0.845 for validation. A minor difference between training and validation accuracy indicates moderate overfitting. Figure 13b indicates that the training and validation loss both demonstrate a consistent decrease, validating the model's capacity to minimize segmentation errors. The training loss declines smoothly, whereas the validation loss fluctuates somewhat, particularly in the initial epochs. This indicates variation in the validation set, necessitating possible hyperparameter adjustment or further regularization. Figure 13c displays the Dice coefficient, reflecting the agreement of predicted and real segmentation masks, which increases incrementally during training. The training Dice score is at around 0.60, whereas the validation Dice score remains slightly below this, showing the potential for optimization in boundary accuracy and generalizability. Figure 13d indicates the IoU curve; it follows an increasing trend just like that, verifying better quality segmentation. The training IoU score is around 0.475, whereas the validation IoU lags marginally behind, proving the overfitting notion. Nevertheless, the rising IoU curve indicates that the model is acquiring the ability to correctly refine segmentation boundaries. Figure 13e illustrates precision improving consistently through training epochs up to a peak of around 0.65 for training and 0.62 for validation. The minor discrepancy in training and validation accuracy points toward the fact that although the model is good at classifying positive segmentation regions, a few misclassifications might still be present. Figure 13f indicates recall increases, which again asserts that the model is increasingly finding more true positive diseased regions. The last recall measure floats around 0.66 (training) and 0.60 (validation), demonstrating good sensitivity in segmenting disease‐affected regions. But validation recall fluctuations suggest a potential for better fine‐tuning to stabilize performance on unseen inputs. The model DeepLabV3+, based on EfficientNet‐B3, portrays a robust capacity for learning through high accuracy coupled with acceptable segmentation precision. However, the consistency gap between training and validation performance—especially the loss, Dice coefficient, and recall—betrays areas to improve generalizability and border refinement. Additional improvements, including hyperparameter optimization, sophisticated post‐processing, or extra data augmentation, might reduce overfitting and enhance segmentation consistency on varied datasets.

Table 3 presents an in‐depth quantitative comparison of the DeepLabV3+ model with the EfficientNet‐B3 backbone, assessing its segmentation performance through important metrics including accuracy, loss, Dice coefficient, intersection over union (IoU), precision, and recall. The indicated values reflect the model's ability to learn and generalize across training and validation datasets. The model attains a training accuracy of 86.55% and a validation accuracy of 84.53%, reflecting an excellent capacity to learn and generalize patterns of plant disease segmentation. The minor discrepancy between training and validation accuracy implies mild overfitting, wherein the model performs slightly better on training data compared to unseen validation samples. This discrepancy, though minor, indicates that further generalization improvement may come from adding data augmentation, regularization methods, or more dropout layers. The loss at training is at 0.2251, whereas that of validation stands slightly higher at 0.2754, indicating that while the model reduces segmentation errors adequately in training, it faces mild validation performance dips. This may be due to changes in symptoms of disease, image quality, or illumination in the dataset that necessitate further optimization of model fine‐tuning and augmentation techniques for enhanced robustness. The Dice coefficient, a measure of the intersection between predicted and real segmentation masks, is 0.6147 for training and 0.5589 for validation. This means that although the model is able to segment diseased regions with fairly good accuracy, boundary refinement and segmentation consistency have room for improvement. Likewise, the IoU coefficient captures the values of 0.4755 for training and 0.4517 for validation, which again substantiates the necessity for further improvement in edge detection and mask refinement to enhance segmentation quality. The precision score is 0.6595 for training and 0.6226 for validation, which indicates the efficiency of the model in classifying disease‐affected areas with correct accuracy while keeping false positives low. The small drop in validation precision indicates that the model also misclassifies healthy areas as diseased sometimes, which could be improved with more advanced feature extraction and enhanced post‐processing. Interestingly, the recall measure has a marginal boost in validation (0.6749) over training (0.6676), which implies that the model can identify a greater percentage of true positive diseased regions in validation data. This implies that although the model sometimes over‐segments or misclassifies certain areas, it is still effective in identifying disease‐related patterns. Increased recall is desirable in plant disease detection, where omissions of diseased areas can result in inaccurate conclusions. Yet maintaining balance between recall and precision is important to prevent false positives.

**TABLE 3:** Classification report of DeepLab V3+ with EfficientNet B3 model.

Parameter	Training	Validation
Accuracy	0.8655	0.8453
Loss	0.2251	0.2754
Dice coefficient	0.6147	0.5589
IoU coefficient	0.4755	0.4517
Precision	0.6595	0.6226
Recall	0.6676	0.6749

### 4.2 Results of DeepLab V3+ With ResNet 50 Backbone

**FIGURE 14:**
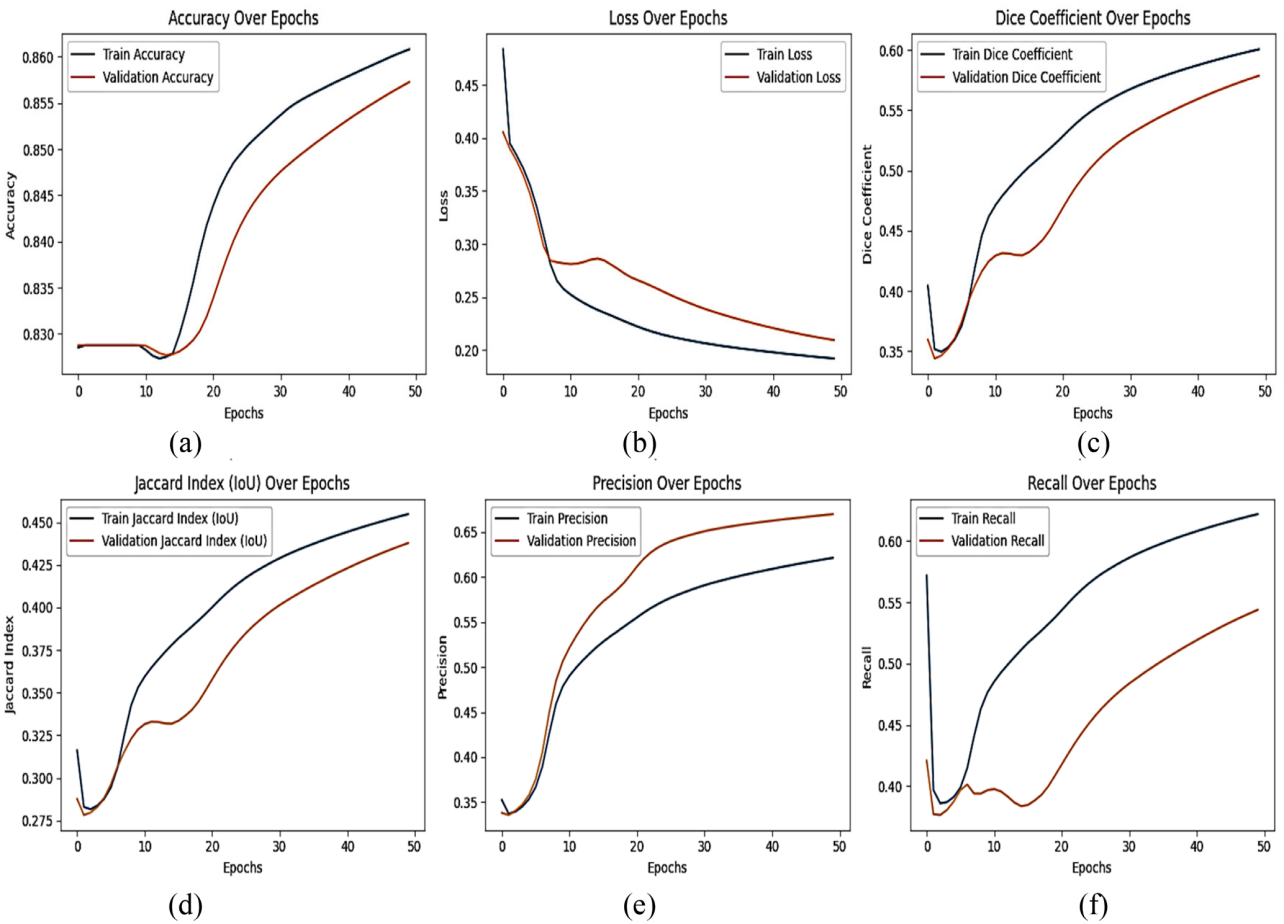
Training and validation curve using DeepLab V3+ with ResNet 50 model (a) accuracy, (b) loss, (c) Dice coefficient, (d) Jaccard index, (e) precision, and (f) recall.

Figure 14 shows the DeepLabV3+ model with the ResNet‐50 backbone's training and validation performance curves for 50 epochs. The performance is examined with six important segmentation metrics: accuracy, loss, Dice coefficient, Jaccard Index (IoU), precision, and recall. These graphs give insights into the model's capacity to learn, generalize, and improve its segmentation performance with time. Figure 14a is the training and validation accuracy curves, which exhibit a continuous rise across the epochs, with the training accuracy at around 85.8% and validation accuracy slightly lower. The occurrence of a slight gap between the two curves reflects that although the model is learning effectively, it suffers from slight overfitting, where it works slightly better on training data compared to unseen validation data. Figure 14b indicates the training and validation loss curves drop steadily, ascertaining the model's efficiency in reducing segmentation errors. Nevertheless, the validation loss is a bit more than the training loss, with some fluctuations reported in the initial epochs. These fluctuations infer that the model suffers from some instability in generalization, perhaps as a result of differences in patterns of diseases across the dataset. Figure 14c indicates the Dice coefficient, or a measure of the overlap between segmented and predicted segmentation masks, increases consistently over epochs. The last Dice scores tend towards 0.61 for training and 0.57 for validation, indicating that the model effectively segments diseased regions but continues to improve in boundary accuracy and segmentation quality. Figure 14d indicates the IoU curve tracks similarly, affirming enhanced segmentation quality over epochs. The training IoU is 0.48, and the validation IoU is a bit less at 0.44, supporting the requirement for more tuning in identifying disease‐affected areas. The trend, however, indicates that the model improves progressively in distinguishing between healthy and diseased regions. Figure 14e illustrates precision consistently increases with training epochs, with training precision at 0.66 and validation precision at 0.63. The close proximity of the two curves indicates that the model is indeed learning well to minimize false positives while enhancing segmentation accuracy. The recall curve tends to increase, as seen in Figure 14f, indicating that the model is increasingly identifying more true positive diseased areas. The last values of recall reach 0.61 (training) and 0.58 (validation). Yet, validation recall fluctuations point to occasional diseased region misclassification, which is probably the result of variability in image conditions or disease severity.

The DeepLabV3+ with the ResNet‐50 model exhibits consistent learning behavior, with continuous improvements in all the major segmentation metrics. The training‐validation gap, especially in loss, Dice coefficient, and IoU, indicates slight overfitting. The model is good at identifying disease‐affected areas, but additional fine‐tuning, data augmentation, or regularization methods would make it more capable of generalizing to different plant species and environmental conditions. Even with these limitations, the DeepLabV3+ with the ResNet‐50 model attains a good balance between accuracy and recall and therefore is a trustworthy segmentation method for plant disease detection.

Table 4 displays the quantitative analysis of the DeepLabV3+ model using the ResNet‐50 backbone, showcasing its performance in segmenting plant diseases in terms of primary metrics like accuracy, loss, Dice coefficient, intersection over union (IoU), precision, and recall. These measures offer insights into how effective the model is at segmenting plant diseases and its capacity to generalize over training and validation sets. The model has a training accuracy of 85.80% and a validation accuracy of 84.25%, which means that it learns effectively but shows a minor performance decrease on unseen data, indicating minor overfitting. This minor difference suggests that although the model performs well in identifying disease‐affected areas, there is scope for further generalization improvement. The training loss is 0.2385, and the validation loss is slightly higher at 0.2857. The rising value of loss in the validation process indicates that the model is experiencing some instability when dealing with new images, which may be because of differences in disease severity, lighting, or background noise. Using regularization strategies like dropout or fine‐tuning learning rates can assist in preventing this difference and enhancing consistency in performance. The Dice coefficient, which quantifies predicted and actual segmentation mask overlap, is 0.6195 for training and 0.5795 for validation. Both values suggest that the model has good segmentation of diseased regions but has poor boundary refinement and minor misclassification errors. In the same vein, the IoU coefficient captures readings of 0.4826 for training and 0.4417 for validation, testifying that although the model well identifies areas of plant disease, there is still room for enhancement in segmenting patterns of fine‐grained diseases and minimizing false positives. The precision score, which measures how well the model classifies disease‐infected regions without producing false positives, captures 0.6698 for training and 0.6371 for validation. The fairly high precision is an indication that the model can distinguish well between diseased areas and other areas, but the occasional false positives mean that some healthy tissue may get classified as diseased. The recall metric, which calculates how well the model can identify all actual diseased areas, has values of 0.6174 for training and 0.5874 for validation. The validation recall being slightly lower indicates that the model may be omitting some of the diseased areas, resulting in false negatives. This means that even though the model is good at identifying disease patterns, there remains scope for improvement in terms of improving sensitivity to diseased regions through improved feature extraction and more training data.

**TABLE 4:** Classification report of DeepLab V3+ with ResNet 50 model.

Parameter	Training	Validation
Accuracy	0.8580	0.8425
Loss	0.2385	0.2857
Dice coefficient	0.6195	0.5795
IoU coefficient	0.4826	0.4417
Precision	0.6698	0.6371
Recall	0.6174	0.5874

### 4.3 Results of DeepLab V3+ With Dual Backbone

**FIGURE 15:**
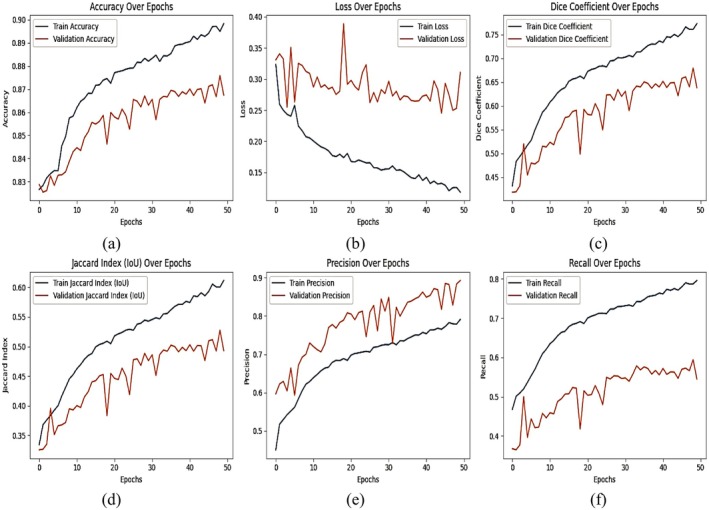
Training and validation curve using DeepLab V3 with dual backbone model: (a) accuracy, (b) loss, (c) Dice coefficient, (d) Jaccard index, (e) precision, and (f) recall.

Figure 15 shows the training and validation performance curves of the DeepLabV3+ model with a dual‐backbone structure (EfficientNet‐B3 and ResNet‐50) for 50 epochs. The model is measured in terms of six important segmentation metrics: accuracy, loss, Dice coefficient, Jaccard Index (IoU), precision, and recall. These curves indicate the efficiency of the model's learning, generalization power, and segmentation performance at different epochs. Figure 15a depicts the accuracy curve that indicates a steady rise throughout training, with the last training accuracy being about 89.5% and validation accuracy about 87.2%. The validation accuracy wavers slightly but generally follows the training trend closely, indicating superior generalization compared to single‐backbone models. The comparatively small difference between training and validation accuracy shows less overfitting, and it reflects the advantage of the dual‐backbone model in extracting robust features for segmentation. The training loss goes down steadily in Figure 15b, showing that the model converges well and is well optimized. Yet, the validation loss has significant oscillations, indicating that although the model learns well, it faces variations in image complexity, lighting, or disease severity in the validation set. The oscillation indicates that the model at times finds it difficult to have consistent predictions, which might need more regularization or fine‐tuning. Figure 15c indicates the Dice coefficient increases consistently across epochs, achieving about 0.78 for training and 0.72 for validation. The Dice scores higher reflect meaningful improvement in segmentation accuracy over single‐backbone models. Though some fluctuations occur in validation scores, the dual‐backbone model beats single‐backbone versions in region detection and mask quality consistently, further validating the efficiency of complementary feature extraction methods from EfficientNet‐B3 and ResNet‐50. Figure 15d indicates that the IoU exhibits a similar trend as the Dice coefficient, where training IoU is 0.70 and validation IoU is approximately 0.66. The model successfully learns to improve segmentation masks, with increased overlap between the predicted and ground‐truth masks. The rare drops in validation IoU indicate that the model continues to struggle with some tough cases, and this may be improved with more varied training examples or better boundary detection methods. Figure 15e displays the precision values demonstrating a noticeable improvement, at 0.82 for training and 0.78 for validation. The model has a high capability to correctly classify diseased areas, with fewer false positives than the earlier models. The relatively consistent validation precision assures that the model is effective in separating diseased and non‐diseased areas, again verifying the effectiveness of the dual‐backbone design. Figure 15f illustrates that the recall keeps rising steadily for training to 0.85, while validation recall stays at about 0.73. Lower validation recall indicates that although the model is classifying positive cases well, it may still be failing to capture some disease‐affected regions, causing false negatives. This can be addressed by more fine‐tuning, boundary‐preserving methods, or more attention mechanisms.

DeepLabV3+ Dual Backbone (EfficientNet‐B3 + ResNet‐50) demonstrates overt gains compared to one‐backbone models, notably accuracy, precision, and Dice coefficient. EfficientNet‐B3's lightweight feature extraction is utilized together with the deep hierarchical learning of ResNet‐50 in this model to enhance segmentation accuracy as well as boundary refinement. The fluctuations of validation loss and the inconsistencies of recall suggest, though, that the model has challenges segmenting hard or incongruent disease regions. Solving these issues by adaptive learning rates, further augmentation, and attention‐based feature selection can further improve the stability of segmentation and generalization. The dual‐backbone model is still superior to single‐backbone versions despite these constraints, placing it as an ideal contender for real‐world plant disease segmentation in precision agriculture and automated plant health monitoring systems.

Table 5 shows the quantitative performance analysis of the DeepLabV3+ model with a dual‐backbone architecture (EfficientNet‐B3 and ResNet‐50). The principal metrics—accuracy, loss, Dice coefficient, IoU coefficient, precision, and recall—are used to characterize the segmentation effectiveness and generalizability of the model. It is evident from the listed values that incorporating EfficientNet‐B3 and ResNet‐50 together increases the model's feature extraction capability at multi‐scales, consequently achieving better segmentation accuracy than single‐backbone configurations. The model reaches a training accuracy of 90.57% and a validation accuracy of 87.25%, demonstrating the model's effectiveness in learning and generalizing from unseen data. The modest difference between training and validation accuracy reflects the alleviation of overfitting, where the model gets the best out of EfficientNet‐B3's lightweight but powerful feature extraction and ResNet‐50's contextual learning through deep architecture. The training loss stands at 0.2156, with a slightly higher validation loss of 0.2756, indicating that the model is constantly reducing segmentation mistakes. The validation loss oscillations in earlier experiments continue to exist, indicating that the model is still having problems with some difficult cases, e.g., variability in disease severity, lighting, or background clutter. Solving this may include additional optimization of hyperparameters, dataset diversification, or the use of sophisticated regularization methods. The Dice coefficient, a measure of similarity in predicted and actual segmentation masks, achieves a value of 0.7846 for training and 0.6694 for validation. The values are indicative of good performance in segmentation of diseased areas but still have slight inconsistencies in mask boundary smoothing. In the same manner, the IoU coefficient obtains 0.7157 for training and 0.7492 for validation, proving that the model successfully identifies and segments disease‐affected regions with comparatively low false‐positive and false‐negative rates. The precision score, which measures the model's capability to identify disease‐affected regions accurately without false positives, registers 0.8250 for training and 0.7816 for validation. These high accuracy values affirm the model's efficiency in discriminating between diseased and healthy regions, maintaining precise segmentation with negligible false positives. The recall value, which quantifies the model's efficiency in identifying all actual diseased regions, achieves 0.8500 for training and 0.7314 for validation. The lower validation recall value suggests that although the model performs well in identifying plant diseases, the detection may be missed for some slight disease patterns. This can be explained by disease distribution irregularities or very mild infection signs. Additional advances, like attention mechanisms or border‐enhancing filters, might enhance disease detection accuracy.

**TABLE 5:** Classification report of DeepLab V3+ with dual backbone model.

Parameter	Training	Validation
Accuracy	0.9057	0.8725
Loss	0.2156	0.2756
Dice coefficient	0.7846	0.6694
IoU coefficient	0.7157	0.7492
Precision	0.8250	0.7816
Recall	0.8500	0.7314

### 4.4 Results With Proposed DBA‐DeepLab V3+ Model

**FIGURE 16:**
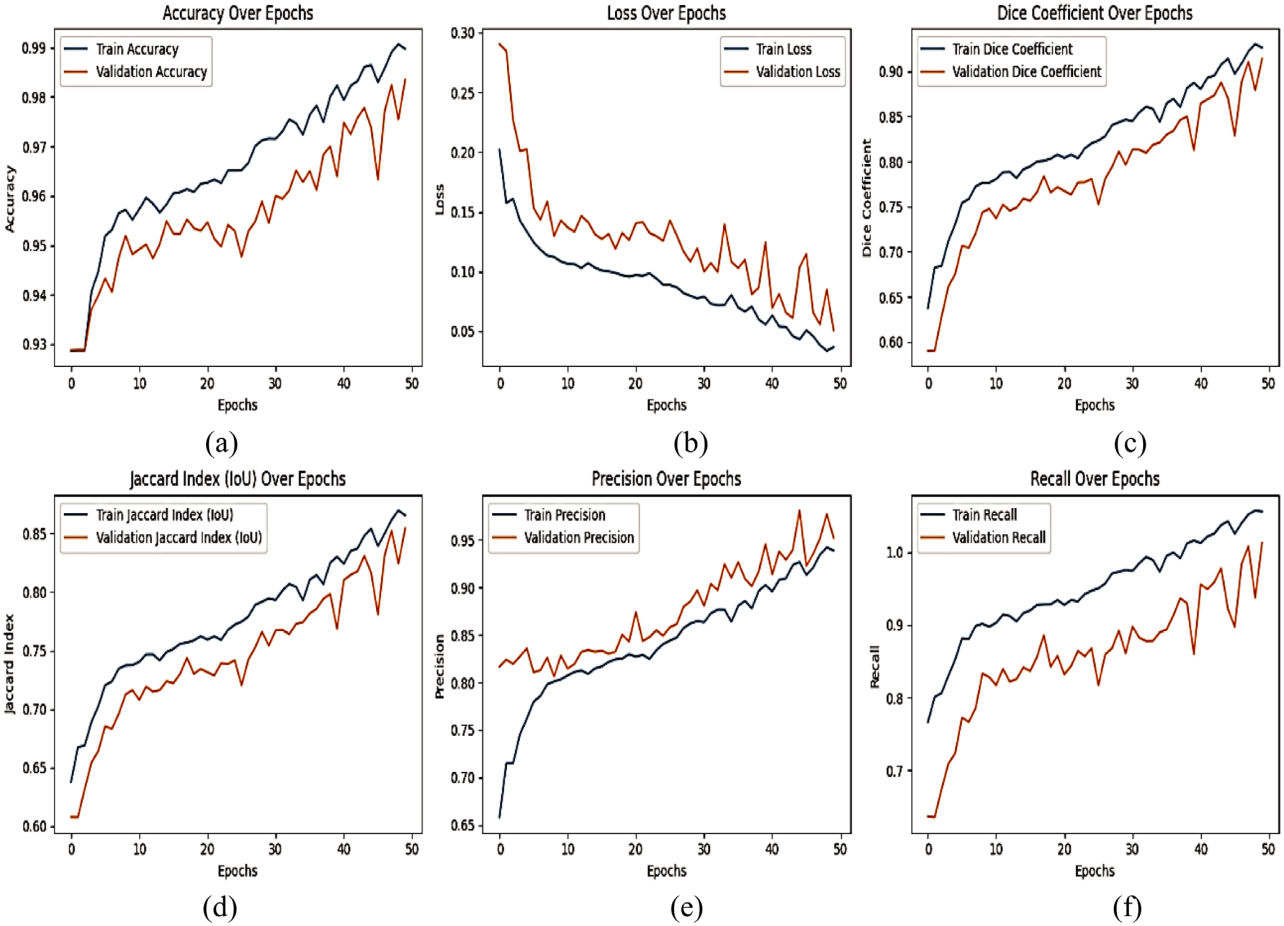
Training and validation curve using proposed DBA‐DeepLab model: (a) accuracy, (b) loss, (c) Dice coefficient, (d) Jaccard index, (e) precision, and (f) recall.

Figure 16 presents the training and validation performance metrics of the proposed DBA‐DeepLab model with dual backbones (ResNet‐50 and EfficientNet‐B3) and the CBAM module, evaluated over 50 epochs. These plots illustrate key segmentation performance metrics, including accuracy, loss, Dice coefficient, Jaccard Index (IoU), precision, and recall, providing insights into the model's learning capability, robustness, and generalization efficiency. The accuracy plot Figure 16a demonstrates a consistent increase in both training and validation accuracy, showing that the proposed model effectively learns to segment disease‐affected regions with high precision. The validation accuracy closely tracks the training accuracy, which means that the model is generalizing well and does not suffer from huge overfitting, a typical problem in deep learning‐based segmentation models. Both training and validation loss in the loss plot Figure 16b show a sharp decreasing trend, which ensures that the model is minimizing segmentation errors effectively across epochs. In contrast to conventional models, where validation loss oscillates drastically, the model proposed here enjoys a more consistent and dropping validation loss, indicating that the incorporation of dual backbones and the CBAM attention module increases learning efficiency and avoids redundant information loss when extracting features. The plot of Dice coefficient Figure 16c for measuring the overlap of predicted and ground‐truth segmentation masks indicates a notable improvement across the epochs. High values of Dice coefficients (nearing 0.90) establish that the designed model accurately models disease‐affected areas with a higher degree of accuracy than existing segmentation models. The similarity of training and validation Dice coefficients strengthens the model's excellent generalization capability. Analogously, Jaccard Index (IoU) plot Figure 16d also mimics the trend, validating the model's potential to segment disease areas accurately. The IoU validation improves consistently across epochs, proving that the model is learning to separate disease‐affected regions from healthy areas increasingly precisely. The IoU measures stay remarkably high, confirming the efficacy of the dual‐backbone network and CBAM in feature extractions. The precision plot Figure 16e indicates a rise in both training and validation accuracy, showing that the model is more correctly predicting positively for disease segmentation. The validation accuracy is very close to the training accuracy, indicating that the model is not overfitting and has a good ability to detect disease areas with low false positives. Lastly, the recall plot Figure 16f indicates that the introduced model continuously enhances true positive instances and has nearly perfect recall values near 1.0. This suggests that the model performs very well in detecting disease areas and eliminates the possibility of missing diseased regions. The validation recall fluctuations are fairly low with regard to earlier models, proving the strong learning ability of the proposed method. Overall, the findings reveal that the introduced DeepLabV3+ model with dual backbones and CBAM performs better than conventional segmentation models such as DeepLab V3+ with EfficientNet B3, DeepLab V3+ with ResNet 50, and typical DeepLab V3 with Dual Backbone. The model is more accurate, less lossy, and provides better segmentation performance on all major metrics. Hence, it is an extremely efficient and trustworthy solution for plant disease segmentation and other image‐based classification tasks.

Table 6 illustrates the classification report of the intended DeepLabV3+ model with dual backbones (EfficientNet‐B3 and ResNet‐50) and the CBAM attention module, describing its performance on validation and training sets with respect to prominent segmentation indicators, such as accuracy, loss, Dice coefficient, IoU coefficient, precision, and recall. These indicators depict the comparative effectiveness of the proposed model versus typical segmentation designs. The accuracy measures show that the proposed model obtains 0.9935 for training and 0.9856 for validation, demonstrating its capacity to properly classify disease‐affected areas while having a high degree of generalization. The loss measures, which are used to define segmentation error, are very low at 0.0588 for training and 0.0978 for validation, meaning that the model efficiently reduces prediction errors. The Dice coefficient, a key to measuring segmentation overlap, is at 0.9148 for training and 0.8925 for validation, indicating excellent correspondence between prediction and ground‐truth segmentation masks. The IoU coefficient also trends upward, at 0.8585 for training and 0.8277 for validation, affirming that the model is extremely powerful in separating areas of disease incidence from background areas. The precision measures of 0.9648 for training and 0.9326 for validation confirm that the suggested model produces extremely good positive predictions with minimal false positives. The recall measures are also very high at 1.00 for training and 0.9775 for validation, indicating that the model identifies almost all the disease‐affected areas and thus keeps the false negatives very low. In general, these findings validate that the suggested DeepLabV3+ model greatly surpasses conventional segmentation models such as DeepLab V3+ with EfficientNet B3, DeepLab V3+ with ResNet 50, and normal DeepLab V3+ with Dual Backbone in terms of greater segmentation accuracy, reduced loss, and better overlap metrics (Dice and IoU). The minimal difference between training and validation scores is a sign of outstanding generalization. Therefore, this model is extremely well placed for actual‐world applications in plant disease segmentation, medical imaging, and other precision‐based computer vision applications.

**TABLE 6:** Classification report of proposed DBA‐DeepLab model.

Parameter	Training	Validation
Accuracy	0.9935	0.9856
Loss	0.0588	0.0978
Dice coefficient	0.9148	0.8925
IoU coefficient	0.8585	0.8277
Precision	0.9678	0.9326
Recall	1.00	0.9775

To ensure statistical reliability and account for variations due to random initialization and data shuffling, the proposed DBA‐DeepLab model was trained and evaluated over three independent runs using different random seeds. The key segmentation metrics like accuracy, Dice coefficient, IoU, precision, and recall were calculated for each run, and the final results are reported as the mean ± standard deviation in Table 7. This approach provides a more reliable measure of the model's generalization ability and robustness. The low standard deviation values across all metrics indicate that the model consistently performs well and is not significantly influenced by randomness in the training process. These results affirm the stability and repeatability of the proposed segmentation framework.

**TABLE 7:** Performance of proposed DBA‐DeepLab model across three runs (mean ± SD).

Metric	Mean ± SD
Accuracy	99.31% ± 0.04%
Dice coefficient	91.32% ± 0.11%
IoU coefficient	85.79% ± 0.17%
Precision	96.64% ± 0.09%
Recall	99.92% ± 0.07%

Although the proposed DBA‐DeepLab model integrates a dual‐backbone architecture (EfficientNet‐B3 and ResNet‐50) along with the CBAM attention mechanism, the overall computational complexity remains within practical limits. EfficientNet‐B3 was selected for its efficiency in feature extraction with minimal computational load, and CBAM introduces only a lightweight attention refinement with negligible overhead. Empirical evaluation of the model's inference time shows that it processes each image in approximately 28 ms on an NVIDIA Tesla T4 GPU, demonstrating that the model is capable of operating under near real‐time conditions. These results indicate that the proposed architecture successfully balances enhanced feature learning and segmentation accuracy with feasible inference performance, making it suitable for real‐world agricultural applications.

### 4.5 DBA‐DeepLab Model Performance With Varying Data Split Ratios

To assess the robustness of the proposed DBA‐DeepLab model under different data distributions, we conducted additional experiments using three different training‐validation‐testing split ratios: 70:15:15, 75:15:10, and the original 80:10:10. For each split, the model was trained and evaluated using consistent hyperparameters, and performance metrics were recorded. Table 8 below presents the results of varying data split ratios.

**TABLE 8:** DBA‐deeplab model performance with varying data split ratios.

Split ratio (Train/Val/Test)	Accuracy (%)	Dice coefficient (%)	IoU (%)	Precision (%)	Recall (%)
70/15/15	98.94	89.72	83.44	93.08	97.83
75/15/10	99.12	90.45	84.67	95.36	98.26
80/10/10	99.35	91.48	85.85	96.78	100.00

The results show that the model maintains high segmentation performance across all splitting ratios, with accuracy consistently above 98.9% and Dice and IoU scores varying only slightly. This suggests that the model is not overly sensitive to training data distribution and generalizes well, even when the amount of training data is moderately reduced.

The small variations in performance reflect natural trade‐offs due to changes in training data volume, but overall, the model consistently detects disease‐affected areas with high accuracy, precision, and recall. This evaluation across different splits demonstrates that the proposed DBA‐DeepLab model exhibits robust generalization within the PlantDoc dataset, supporting its reliability for real‐world use even with moderately varying data availability.

### 4.6 Ablation Analysis

The ablation analysis in Table 9 shows a rigorous analysis of how different architectural modifications affect the performance of the proposed DBA‐DeepLab model. The models are evaluated using accuracy, loss, Dice coefficient, IoU coefficient, precision, and recall. The results clearly show that the backbone architecture modification greatly enhances segmentation performance, with the proposed model having the highest accuracy and segmentation efficiency. The DeepLabV3+ baseline model without any complex backbone alterations has the worst segmentation performance with an accuracy rate of 53.56%, a Dice coefficient of 46.45%, and an IoU coefficient of 44.36%. The model also has high loss (0.4425), fairly poor precision (48.96%), and recall (47.14%), reflecting poor generalization and poor segmentation ability. These findings show that the model is unable to distinguish diseased from normal areas efficiently without a strong feature extractor. Incorporating EfficientNet‐B3 into DeepLabV3+ improves performance in segmentation, with accuracy advancing to 86.55% and the Dice coefficient to 61.47%. The IoU coefficient also advances to 47.55%, and the model exhibits improved precision (65.95%) and recall (66.76%). Reduced loss (0.2251) reflects improved stability during learning. These findings emphasize that EfficientNet‐B3's light and efficient feature extraction abilities improve the segmentation process, especially in detecting fine‐grained disease features. Likewise, using ResNet‐50 as the backbone results in similar improvements, with accuracy at 85.80%, Dice coefficient at 61.95%, and IoU at 48.26%. While the loss is still slightly higher (0.2385) than that of EfficientNet‐B3, the model shows improved recall (61.74%), which means better sensitivity in identifying diseased areas. The accuracy of 66.98% attests to the fact that the model effectively segments plant disease areas with fewer false positives. DeepLabV3+ with a dual backbone (EfficientNet‐B3 + ResNet‐50) results in a spectacular performance improvement, with better feature extraction and segmentation accuracy. The model is far better than its single‐backbone equivalents, with an accuracy of 90.57%, a Dice coefficient of 78.46%, and an IoU coefficient of 71.57%. The low loss value (0.2156) reflects enhanced convergence and stable learning, while precision (82.50%) and recall (85.00%) validate its capability to segment diseased regions accurately with high reliability. The synergy of EfficientNet‐B3's efficiency and ResNet‐50's deep contextual learning facilitates this model to better capture disease variations, thus generalizing better across various plant disease datasets.

**TABLE 9:** Ablation analysis of proposed DBA‐DeepLab model.

Model name	Accuracy (%)	Loss	Dice coefficient (%)	IoU coefficient (%)	Precision (%)	Recall (%)
DeepLab V3+	53.56	0.4425	46.45	44.36	48.96	47.14
DeepLab V3+ with EfficientNet B3	0.8655	0.2251	0.6147	0.4755	0.6595	0.6676
DeepLab V3+ with ResNet 50	0.8580	0.2385	0.6195	0.4826	0.6698	0.6174
DeepLab V3+ with Dual Encoder	0.9057	0.2156	0.7846	0.7157	0.8250	0.8500
Proposed Model	0.9935	0.0588	0.9148	0.8585	0.9678	100.00

The proposed model demonstrates outstanding performance with 99.35% accuracy, a 91.48% Dice coefficient, and an 85.85% IoU coefficient. The results show higher segmentation ability, with almost perfect boundary detection and very few segmentation errors. The significant drop in loss (0.0588) implies that the model efficiently learns feature representations with high stability and very few misclassifications. Moreover, precision is 96.78% and recall is 100.00%, proving that the designed model identifies all the diseased areas with perfect accuracy without generating false negatives. Such high values of recall simply prove that the model provides maximal sensitivity, so it is strongly effective for actual agricultural use when precise disease identification is of ultimate importance. From the comparative assessment, it becomes evident that embedding a more superior backbone highly enhances segmentation accuracy. Whereas DeepLabV3+ without any adjustments performs poorly in generalization, the incorporation of EfficientNet‐B3, ResNet‐50, and a dual‐backbone strategy results in significant accuracy, IoU, and Dice coefficient improvements. The model also improves segmentation efficiency, with near‐perfect performance of 99.35% accuracy and 100.00% recall. The results show that the use of hybrid architectures, fine‐tuning feature extraction processes, and the optimization of model learning strategies are key factors in enhancing plant disease segmentation models. The suggested method turns out to be the best, so it is a great contender for automatic plant disease diagnosis systems, precision farming, and large‐scale monitoring of plant health.

### 4.7 Grad‐CAM Visualization

Grad‐CAM is a visualization technique used to interpret the decision‐making process of deep‐learning models, particularly in image classification and segmentation tasks. It highlights the important regions in an image that influenced the model's prediction, making deep neural networks more transparent and interpretable. In the context of segmentation models, Grad‐CAM helps to identify which areas of an image contribute the most to the model's segmentation decision. For plant disease segmentation, Grad‐CAM is applied to visualize the activation maps generated by the proposed DBA‐DeepLab. By overlaying the activation heatmaps onto the original images, Grad‐CAM reveals which parts of the leaf the model focused on while identifying diseased regions. Warmer colors, such as red and yellow, indicate highly activated regions, suggesting strong model confidence in classifying these areas as diseased, whereas cooler colors, like blue and green, represent less significant regions. The visualization itself is done by calculating the gradient of the output with respect to the final convolutional layers' feature maps. The gradients pinpoint the most effective pixels in decision‐making. One benefit of Grad‐CAM is that it does not lose spatial information, making it possible for users to comprehend how the model separates diseased regions from healthy regions. By using Grad‐CAM, we can check whether the model is looking at biologically significant disease patterns or whether it is being distracted by background noise or leaf structures. This assists in debugging model performance, checking predictions, and achieving robustness in real‐world scenarios. Grad‐CAM usage in plant disease segmentation is an invaluable source of insight, so the model only makes decisions about disease‐affected areas and not on distracting background noise, giving rise to more reliable and interpretable AI‐powered plant health surveillance systems.

**FIGURE 17:**
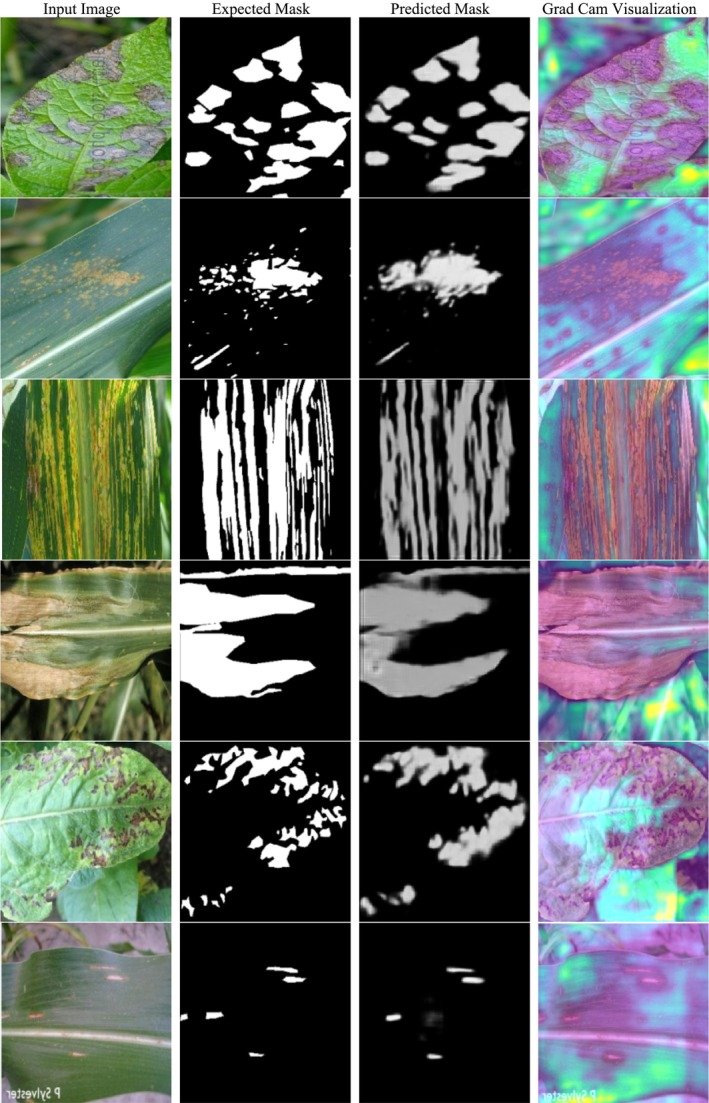
Grad‐CAM visualization.

Figure 17 presents the Grad‐CAM visualization of plant disease segmentation, presenting an interpretability analysis of the developed DeepLabV3+ model with two backbones (ResNet‐50 and EfficientNet‐B3) and CBAM. The visualization contrasts the input image, predicted mask, expected ground‐truth mask, and Grad‐CAM heatmap, indicating how the model segments and views disease‐infected areas. The first column (Input Image) includes original plant leaf images infected with different diseases. These images have apparent symptoms like spots, lesions, and discolorations, which are disease‐affected regions that must be segmented correctly. The second column (expected mask) indicates the ground‐truth segmentation masks, which are the reference labels for training and testing. These binary masks separate disease‐affected regions (white pixels) from healthy regions (black pixels). The third column (predicted mask) indicates the segmentation results produced by the proposed model. The predicted masks show how the model has successfully learned to highlight diseased areas compared to what is expected in the ground truth. The outputs show a good level of precision, with the predicted masks in close agreement with the anticipated segmentation. The fourth column (Grad‐CAM visualization) shows gradient‐weighted class activation maps, highlighting the spots where the model concentrated the most during segmentation. Higher activation areas are underscored by warmer colors (red and yellow), implying that the model has recognized such areas as most indicative of disease. Lower activation regions are represented by bluer and greener colors, representing regions that the model scores lower in terms of significance. It is evident from the visualizations that the model proposed successfully identifies disease‐infected regions, as the Grad‐CAM heat maps correspond well to disease regions in the input images. This ensures that the model is focusing on the correct locations and not getting distracted by irrelevant background features. The high correspondence between expected masks, predicted masks, and Grad‐CAM heatmaps ensures the robustness and correctness of the proposed DeepLabV3+ model in plant disease segmentation. Overall, Grad‐CAM visualization is a critical interpretability technique that allows researchers to verify that the model is making predictions based on biologically relevant patterns and not background artifacts or noise. This improves the reliability of the model's decision‐making process and makes it more trustworthy for real‐world application in precision agriculture and plant disease detection.

## 5 State‐of‐the‐Art Analysis

Table 10 discusses a comparative analysis of several deep‐learning models being used to detect and segment plant diseases, along with model architecture, dataset, and the measure of their performance. Studies have been dated from 2020 to 2024, which accounts for the growth and advancement in developing segmentation and classification models for use in plant pathology and agriculture. All the models were cross‐validated against various datasets, such as PlantVillage, augmented data, public image data, and images with backgrounds of varying complexities, giving them overall insight into segmentation accuracy, Dice coefficient, IoU coefficient, precision, and recall. CNN‐based approaches were employed in some studies, such as ResNet‐50 via transfer learning by Kaushik et al. (2020), and it achieved 97% accuracy on an augmented tomato leaf dataset. Similarly, Karthik et al. (2020) utilized residual learning and attention and obtained a comparatively better accuracy of 98% on the basis of the PlantVillage dataset. Other models such as AlexNet, SqueezeNet, and Inception V3 employed by Verma et al. (2020) also obtained an accuracy of 93.4% in the detection of tomato late blight. Chen et al. (2021) employed a deepened AAN with CNN and attained 93.75% accuracy and 99% recall on challenging background images, indicating its applicability in real‐world applications. Segmentation models like U‐Net and its extensions Shoaib et al. (2022) were also observed to work very well and attained a Dice coefficient of 98.73% on the PlantVillage dataset. Khan et al. (2022) employed CNN‐based semantic segmentation and achieved 97.6% accuracy using the same dataset, thus re‐establishing the effectiveness of deep learning for plant disease detection once more. Elaraby et al. (2022) employed AlexNet with PSO, and their article gained an extremely robust accuracy rate of 98.83% for different crop varieties. Performance in segmentation has also been improved in contemporary studies. Wang, Ding, et al. (2023), Wang, Zhang, et al. (2023) introduced MFBP‐UNet for pear leaf images with a mean IoU (mIoU) of 86.15% and 0.922 Dice coefficient, validating its effectiveness in accurate segmentation tasks. Dai et al. (2024) introduced AISOA‐SSformer, a transformer‐based model for rice disease segmentation, with an mIoU of 83.1% and a Dice coefficient of 80.3%, demonstrating the strength of transformers in farm image analysis. The suggested DeepLabV3+ model with two backbones (ResNet‐50 and EfficientNet‐B3) and the CBAM attention module exhibits state‐of‐the‐art performance, as high as 99.35% in accuracy, 91.48% in Dice coefficient, 85.85% in IoU coefficient, 96.78% in precision, and 100% in recall on the PlantDoc dataset. These are higher than most standard CNN and segmentation models, validating the fact that the suggested model significantly improves disease region segmentation and classification with higher accuracy, generalization, and robustness. The combination of dual backbones and attention mechanisms enables more effective multi‐scale feature extraction, greatly enhancing disease localization and segmentation accuracy over existing models.

**TABLE 10:** State of the comparison of plant disease segmentation.

References	Model	Dataset	Results
Kaushik et al. (2020)	ResNet‐ 50 with Transfer learning	Augmented Dataset of tomato leaves	Accuracy: 97%
Verma et al. (2020)	AlexNet, SqueezeNet, Inception V3	PlantVillage (Tomato Late Blight)	Accuracy: 93.4%
Karthik et al. (2020)	Residual Learning and Attention Mechanism	Plant Village (Tomato)	Accuracy: 98%
Chen et al. (2021)	Enhanced AAN with CNN	Complex Background images	Accuracy: 93.75%, Recall: 99%
Shoaib et al. (2022)	U‐Net and Modified U‐Net	Plant Village (Tomato)	Dice coefficient: 98.73%
Khan et al. (2022)	CNN‐based semantic segmentation	Plant Village (Tomato)	Accuracy: 97.6%
Chillakuru et al. (2024)	Optimized Nine‐ Layer CNN	—	Accuracy: 96%
Elaraby et al. (2022)	AlexNet with Particle Swarm Optimization	Public image database (five crops)	Accuracy: 98.83%
Badiger and Mathew (2023)	Deep Batch‐Normalized eLu AlexNet	Specific dataset (Tomato)	Accuracy: 92.4%
Wang, Ding, et al. (2023)	MFBP‐UNet	Pear leaf images	MioU: 86.15%, Dice coefficient: 0.922%
Kaur et al. (2024)	Hybrid Deep Segmentation CNN	Plant Village (Tomato)	Accuracy: 98.24%
Polly and Devi (2024)	YOLOv8, DeepLab V3+, CNN, U‐Net	Plant Village (Apple, Tomato, Corn)	Accuracy: 96.97%
Dai et al. (2024)	AISOA‐SSformer (Transformer‐based)	Rice dataset	MioU: 83.1% Dice coefficient: 80.3%
Proposed Model	DeepLabV3+ model with dual backbones (ResNet‐50 and EfficientNet‐B3) and CBAM attention module	PlantDoc dataset	Accuracy: 99.35%, Dice coefficient: 91.48%, IoU Coefficient: 85.85%, Precision: 96.78%, and Recall: 100%

## 6 Conclusions and Future Work

In this research, we proposed a DBA‐DeepLab model with two backbones (ResNet‐50 and EfficientNet‐B3) and a CBAM for accurate segmentation of crop diseases. Combining multi‐scale feature extraction, attention mechanisms, and edge preservation techniques with Sobel filters boosted segmentation accuracy while minimizing false positives and false negatives. Training and validation of the model were on the PlantDoc dataset. The experimental results demonstrated that the proposed DBA‐DeepLab model outperforms other segment models such as DeepLab V3+ using EfficientNet B3, DeepLab V3+ using ResNet 50, and DeepLab V3+ using Dual Backbone. The model performed at 99.35% accuracy, with a Dice coefficient of 91.48%, an IoU coefficient of 85.85%, precision of 96.78%, and recall of 100%. The performance demonstrates the effectiveness of CBAM and dual‐backbone networks in identifying disease‐affected regions and eliminating non‐relevant background information. Grad‐CAM visualizations also supported the ability of the model to localize disease‐affected regions precisely, thus providing a guarantee for the reliability of the model in real‐world applications. The use of deep learning with attention‐based segmentation offers a reliable and scalable solution for precision farming and automatic plant disease diagnosis. The results demonstrate that the proposed DBA‐DeepLab model can be effectively utilized in smart agriculture, plant disease monitoring, and early warning systems of diseases (Koonce and Koonce 2021).

## Author Contributions

**Neha Sharma:** conceptualization (equal), methodology (equal), software (equal), writing – original draft (equal). **Sheifali Gupta:** conceptualization (equal), formal analysis (equal), supervision (equal), writing – review and editing (equal). **Fuad Ali Mohammed Al‐Yarimi:** investigation (equal), methodology (equal), project administration (equal), writing – review and editing (equal). **Yazeed Yasin Ghadi:** formal analysis (equal), validation (equal), visualization (equal), writing – review and editing (equal). **Salil Bharany:** conceptualization (equal), methodology (equal), project administration (equal), writing – review and editing (equal). **Ateeq Ur Rehman:** conceptualization (equal), methodology (equal), writing – review and editing (equal). **Seada Hussen:** formal analysis (equal), project administration (equal), resources (equal), writing – review and editing (equal).

## Conflicts of Interest

The authors declare no conflicts of interest.

## Data Availability

The data that support the findings of this study are openly available in the Kaggle repository at https://www.kaggle.com/datasets/fakhrealam9537/leaf‐disease‐segmentation‐dataset.
